# The development of cortical processing of speech differs between children with cochlear implants and normal hearing and changes with parental singing

**DOI:** 10.3389/fnins.2022.976767

**Published:** 2022-11-18

**Authors:** Ritva Torppa, Soila Kuuluvainen, Jari Lipsanen

**Affiliations:** ^1^Department of Psychology and Logopedics, Faculty of Medicine, University of Helsinki, Helsinki, Finland; ^2^Cognitive Brain Research Unit, Department of Psychology and Logopedics, Faculty of Medicine, University of Helsinki, Helsinki, Finland; ^3^Centre of Excellence in Music, Mind, Body and Brain, Faculty of Medicine, University of Helsinki, Helsinki, Finland; ^4^Department of Digital Humanities, Faculty of Arts, University of Helsinki, Helsinki, Finland

**Keywords:** cochlear implant, positive mismatch responses (pMMR), attention-related brain response P3a, mismatch negativity (MMN), late differentiating negativity (LDN), parental singing

## Abstract

**Objective:**

The aim of the present study was to investigate speech processing development in children with normal hearing (NH) and cochlear implants (CI) groups using a multifeature event-related potential (ERP) paradigm. Singing is associated to enhanced attention and speech perception. Therefore, its connection to ERPs was investigated in the CI group.

**Methods:**

The paradigm included five change types in a pseudoword: two easy- (duration, gap) and three difficult-to-detect (vowel, pitch, intensity) with CIs. The positive mismatch responses (pMMR), mismatch negativity (MMN), P3a and late differentiating negativity (LDN) responses of preschoolers (below 6 years 9 months) and schoolchildren (above 6 years 9 months) with NH or CIs at two time points (T1, T2) were investigated with Linear Mixed Modeling (LMM). For the CI group, the association of singing at home and ERP development was modeled with LMM.

**Results:**

Overall, responses elicited by the easy- and difficult to detect changes differed between the CI and NH groups. Compared to the NH group, the CI group had smaller MMNs to vowel duration changes and gaps, larger P3a responses to gaps, and larger pMMRs and smaller LDNs to vowel identity changes. Preschoolers had smaller P3a responses and larger LDNs to gaps, and larger pMMRs to vowel identity changes than schoolchildren. In addition, the pMMRs to gaps increased from T1 to T2 in preschoolers. More parental singing in the CI group was associated with increasing pMMR and less parental singing with decreasing P3a amplitudes from T1 to T2.

**Conclusion:**

The multifeature paradigm is suitable for assessing cortical speech processing development in children. In children with CIs, cortical discrimination is often reflected in pMMR and P3a responses, and in MMN and LDN responses in children with NH. Moreover, the cortical speech discrimination of children with CIs develops late, and over time and age, their speech sound change processing changes as does the processing of children with NH. Importantly, multisensory activities such as parental singing can lead to improvement in the discrimination and attention shifting toward speech changes in children with CIs. These novel results should be taken into account in future research and rehabilitation.

## Introduction

Cochlear implants (CIs) provide hearing for congenitally deaf children. The CI captures external sound with a microphone, processes and changes it to electric signal, and delivers the signal with the electrodes implanted in the inner ear to the auditory nerve of the user. For most children hearing with CIs, the ability to perceive speech is the most important change post-implantation, as it allows oral communication with others. The CIs, however, provide some speech cues well, whereas other speech cues are underrepresented by the device, when compared to the auditory system of normally hearing (NH) individuals. In general, gross temporal changes, such as changes in duration or insertion of gaps in the speech stream, are well delivered by CIs (see Limb and Roy, 2014; Torppa et al., 2014a,b). Changes in vowel duration signal in most languages prosodic word and sentence stress, while gaps are sometimes inserted between words and sentences, and thus these temporal changes can aid in speech segmentation (Vavatzanidis et al., 2015). However, in quantity languages such as Finnish, they can also indicate a change in word meaning (see Suomi et al., 2008). For example, inserting a gap before the /t/ in the Finnish word “muta” (mud) changes it to “mutta” (but) and elongating the /u/ changes it to “muuta” (other). Thus, Finnish CI users will have to use temporal information to capture both semantic and prosodic changes in the speech stream.

In contrast to the relatively well-preserved gross temporal aspects, the signal delivered by CIs lacks detail in spectral and fine temporal information compared to that of typical human hearing (Moore, 2003). This hampers the accurate perception of pitch (fundamental frequency; F0) and timbre (Chatterjee and Peng, 2008; Limb and Roy, 2014; Oxenham, 2018), and the perception of changes in the vowels and consonants in children with CIs (Geers et al., 2003; Donaldson and Kreft, 2006; for a review, see Rødvik et al., 2018). Therefore, the changes from “muta” (mud) to “muna” (an egg) or from “muuta” (other) to “maata” (to lie down) are typically less easily perceived by CI users than the semantic changes based on temporal changes. Moreover, the dynamic range (the range between the detection threshold of sound or electric current and the point where the sensation becomes uncomfortable, Moore, 2003) of CIs is limited compared to NH, harming the perception of changes in intensity (Moore, 2003; Limb and Roy, 2014). Poor pitch and intensity resolution further leads to difficulties in the perception of speech prosody, which is important to children’s speech segmentation and language learning (word and sentence stress: O’Halpin, 2010; Torppa et al., 2014a,^2020^; intonation: Chatterjee and Peng, 2008; Moein et al., 2017) and the perception of emotions (Luo et al., 2007; Hopyan-Misakyan et al., 2009; Chatterjee et al., 2015, 2019; Paquette et al., 2018). The perceptual deficits also lead to increases in listening effort compared to NH, often leading to frustration and poor quality of life in CI users (Dimitrijevic et al., 2019).

The evidence on where or how the cortical processing of children with CIs might differ from that of children with NH is based mainly on indirect evidence, using degraded speech with adults or children with NH. Speech of children is processed by the bilateral temporal cortices and inferior frontal gyrus (Lawrence et al., 2021). When listening of children or adults comes more effortful (as for children with CIs compared to children with NH), in adults, the brain activity seems to increase in the prefrontal cortex, and in children, the speech processing lateralizes more to the left hemisphere (Wild et al., 2012; Evans and Davis, 2015; Lawrence et al., 2021). It thus possible that when listening to speech effortfully, children with CIs utilize cognitive auditory attention and working memory mechanisms, as well as motoric representations of speech.

On the other hand, long-term, congenital sensory deficiencies have been shown to lead to cross-modal brain plasticity, that is, to the recruitment of the auditory cortex by visual or tactile (somatosensory) input, harming the development of cortical networks for speech processing and speech perception (for a review, see Campbell and Sharma, 2016; Glick and Sharma, 2017). Atypical development of the cortical speech areas and their connections, together with the unclear signal from CIs, may harm auditory attention and cognitive processing necessary for speech perception and understanding in children with CIs (for reviews, see Price, 2012; Friederici, 2017; King et al., 2021). Fortunately, children’s brains are plastic, and, if implanted earlier than at the age of 3.5 years, children with CIs acquire fairly good behavioral speech perception and language skills (van Wieringen and Wouters, 2015; Campbell and Sharma, 2016). However, the behavioral observations tell very little on whether the processing is similar to that of NH peers, or if it is affected by higher listening effort and/or compensatory processing. Therefore, research has turned to event-related potentials (ERPs) as psychophysiological markers of the underlying cognitive processes in studying typical and atypical speech perception in both children and adults.

In investigating cortical processing, ERPs, extracted from electroencephalograpy (EEG), can illuminate the reactions of the brain to changes in the auditory environment, even without the listener having to pay attention to the sound (Näätänen et al., 2017, among others). EEG is a non-invasive method, which can be used in small children, even in neonates (see, e.g., Partanen et al., 2013a). The cortical processing of an auditory change is typically reflected in a chain of positive and negative ERP responses, each deflection corresponding to a different aspect of change detection, such as detection, attention call, and further processing (for a review, see Hu et al., 2021).

Perhaps the most widely studied of these responses is the mismatch negativity (MMN), elicited in adults 100–200 ms after stimulus onset for changes in speech sound features (for a review, see Näätänen et al., 2017). In addition, increases in MMN amplitudes and decreases in their latencies are associated with better behavioral change detection abilities (Näätänen et al., 2007, 2017). In NH listeners, MMN amplitudes are maximal in the frontotemporal scalp areas, with generators located bilaterally in the supratemporal auditory cortices, and also in the right frontal cortex (Pihko et al., 2005; Kuuluvainen et al., 2014; for a review, see Näätänen et al., 2007). Compared to the adult MMNs, children’s MMNs are elicited at a wider time window, between 100 and 400 ms after a change in the sound stream, the latency and amplitude varying depending on age, magnitude of change, and stimulus type (Shafer et al., 2010; Paquette et al., 2013; for a review, see Näätänen et al., 2007).

In children, MMN can be replaced with positive mismatch response (pMMR), and sometimes both pMMR and MMN are elicited, with the pMMR preceding or following the MMN (Shafer et al., 2010; Lee et al., 2012). The pMMR was first reported in babies (Dehaene-Lambertz and Dehaene, 1994), but later studies reported pMMRs also in older children, up to the age of 12 years, for changes that are small or difficult to detect (Maurer et al., 2003; Shafer et al., 2010; Lee et al., 2012; Partanen et al., 2013b). Since pMMRs have been found to diminish with children’s age (Morr et al., 2002), and the same stimulus feature change can elicit positive pMMRs in younger NH children but negative MMN responses in older NH children (Shafer et al., 2010), the pMMR may also reflect neural immaturity. As further evidence of this, in NH children aged 4–12 years, individual level incidence of pMMR for vowel changes was associated with poorer performance intelligence quotient (PIQ) scores 14–17 months after the recording (Partanen et al., 2013b).

The MMN has been elicited in both children and adults with CIs (for a review, see Näätänen et al., 2017), and with amplitudes becoming larger with better behavioral perception (for pitch: Cai et al., 2020; for speech: Turgeon et al., 2014; for a review, see Näätänen et al., 2017). Furthermore, the pMMR has also been elicited in children with CIs (for a review, see Ziatabar Ahmadi et al., 2022). For instance, using a multifeatured paradigm (see Näätänen et al., 2017) with harmonic or musical tones, pMMRs were observed in the time window of the MMN (Engström et al., 2020; for “CI singers” in Torppa et al., 2014b,^2018^). Notably, pMMRs in these studies were associated with good rather than with poor perceptual abilities. Further, positive, pMMR-like deflections before MMN can be seen (but were not analyzed) for changes in speech in 7–19 year-old children with CIs who perform well in speech discrimination tasks (Figure 2, CFz electrode, approximately 150 ms, in Ortmann et al., 2013a).

In NH adults and children, the MMN is often followed by the P3a response. The P3a is elicited if the change is clearly detected (see Torppa et al., 2014b; for a review, see Wetzel and Schröger, 2014) and, in the NH population, P3a reflects an attention switch toward a sound change (Friedman et al., 2001; Horváth et al., 2008; Wetzel and Schröger, 2014). Similarly to the MMN, P3a amplitude increases together with increasing difference between the deviant and standard, suggesting that the more salient the change is for the perceiver, the more likely it is to result in an involuntary attention shift (Winkler et al., 1998; Wetzel et al., 2006; Wetzel and Schröger, 2014). In NH, the neural network of the P3a is distributed across frontal, parietal and temporal cortical regions (Takahashi et al., 2013). However, while the auditory MMN generators are typically stronger in the auditory temporal areas, the emphasis in P3a elicitation is in frontal brain areas devoted to attention-related processing (Takahashi et al., 2013). Thus, the P3a is also usually easily detectable from the frontocentral electrode locations, if elicited.

Similarly to pMMR and MMN, the P3a has also been elicited in children with CIs (for a review, see Näätänen et al., 2017). To the best of our knowledge, there is only one study directly assessing P3a to changes in a speech stimulus in children with CIs. Kileny et al. (1997) found that the P3a latency was later but its amplitude larger for speech contrasts (heed vs. who’d) than for frequency changes (1500 vs. 3000 Hz tone bursts) in children with CIs aged between 4 and 12 years. Furthermore, the larger and earlier the P3a for the frequency changes was, the better was the speech recognition (Kileny et al., 1997). The results suggest that in children with CIs, automatic attention shifts to auditory stimuli are important for their speech perception abilities.

The late differentiating negativity (LDN), sometimes also named late MMN, appears often after the pMMR or MMN and P3a responses in NH children (Korpilahti et al., 2001; Bishop et al., 2011; Liu et al., 2014; Kuuluvainen et al., 2016a). The LDN amplitude becomes smaller when the physical change in the stimulus becomes larger, and the LDN appears relatively often in children but is usually absent in adults (Bishop et al., 2011; Kuuluvainen et al., 2014, 2016a; Liu et al., 2014). The functional role of LDN is unclear. It may reflect cognitive processing or additional cognitive processing (“a second look”) of auditory stimuli that are complex or hard to discriminate (Shafer et al., 2005; Bishop et al., 2011), or the ongoing establishment of internal phonological representations in children (Liu et al., 2014). Kuuluvainen et al. (2016a) investigated the relationship of the LDN to several cognitive tasks in 6-year-old children. In this study, better verbal and non-verbal reasoning were associated with declining amplitudes of the LDN to vowel-like changes in complex non-speech sounds, but not to vowel changes in syllables. The results suggest that the appearance of the LDN to irrelevant sound changes indexes poorer cognitive maturity. Thus, it is, to date, unclear whether the elicitation or size of the LDN is a marker of more mature or more immature processing, as it might depend on the stimulus and the change type.

According to the best of our knowledge, the LDN responses for changes in speech have only rarely been found in children with CIs. Singh (2005) studied in his doctoral dissertation 7–17 year-old children with CIs using computer-generated /ba/ and /da/ stimuli. The LDN was found in children with CIs, but only in those children whose auditory performance was poor. Singh also found that the longer was the duration of LDN response, the poorer was the auditory performance and speech intelligibility (Singh, 2005). Hu et al. (2021) studied in Mandarin-Chinese speaking children with NH and CIs, aged 7–13 years, the LDN responses to changes in lexical tones in monosyllable or four-syllable idiom (phrase) conditions. While the MMN was elicited in both child groups in both conditions (being smaller in the idiom context in children with CIs), the LDN was found only in the children with NH (Hu et al., 2021; see also similar results by Uhlén et al., 2017, for harmonical sine tones).

There are only few studies directly addressing the development of cortical processing of speech reflected in the chain of pMMR and/or MMN, and P3a responses in children with CIs, with literature on LDN development lacking completely. Regarding early development, Vavatzanidis et al. (2015) found that already 4 months after the first auditory input (age at CI activation from 11 to 45 months), the MMN amplitudes to changes in vowel duration, representing syllabic stress, became similar in CI children compared to those of the NH group. Vavatzanidis et al. (2016) also followed the development of the MMN of children with CIs for changes in syllable stress patterns, again cued with vowel duration, in trochaic (similar to the native language of the participants) and iambic (not typical for the native language) stimuli. In this study, the age at first activation of the CI was 9–50 months. They found that the ERP waveforms of the children with CIs were very similar to those of their NH peers. For both CI and NH children, a MMN-like mismatch response was present for the “non-native” iambic deviant, but not for the trochaic stimulus, suggesting that familiarity and multisensory learning of native language affects the development of MMN for speech stimuli similarly for CI and NH children (Vavatzanidis et al., 2016).

In addition to the signal for CIs and age at implantation and onset of hearing with CIs (explained above), there are several factors that have been extensively studied and known to improve the speech perception of children using unilateral or bilateral CIs. These factors include early age at diagnosis of the hearing impairment, extensive oral communication, the ability of the electrodes to activate the auditory nerve, and supportive involvement of the parents (for a review, see van Wieringen and Wouters, 2015). One of the most understudied factors that could improve speech perception of children with CIs is singing. The children in the current study have participated in previous studies in which the relationship of the children’s own and their parents’ singing to the children’s perceptual processing was investigated. Those children with CIs who sang regularly (“CI singers”), and whose parents sang regularly for them, were better in perception of speech in noise than other children with CIs (”CI non-singers”; Torppa et al., 2018). Moreover, the more the children with CIs participated in musical activities including singing, the better they perceived word and sentence stress and pitch (F0) changes in synthesized speech syllables (Torppa et al., 2014a). Furthermore, the more parents sang for them, the better they were in word finding and verbal IQ (Torppa et al., 2020). There are several possible reasons for the improvement of speech perception and language skills with singing. For instance, the slower tempo of lyrics in songs compared to the tempo of speech makes it easy to improve speech perception through listening to singing. Moreover, singing by children themselves is a repetitive, multisensory activity, both aspects being important for perceptual learning and the development of neural networks related to speech and language processing (for reviews, see Torppa et al., 2018; King et al., 2021). Above all, singing is known to arise the attention of young children (Corbeil et al., 2013) and keeping it for longer time than speech (Politimou et al., 2019), which is probably highly beneficial for speech processing. Since also children with CIs pay special attention to singing, it is used in their speech and language therapy (Ronkainen, 2011; McConkey Robbins, 2020). Attention toward sounds increases activation in auditory cortex (Fritz et al., 2007; Woods and Alain, 2009; Woods et al., 2009), and it has been emphasized that speech has to be brought directly to the attention of children with hearing impairments, to make them aware of sounds and speech (for instance Cole and Flexer, 2019, p. 189). Thus, there are several reasons why parental singing could improve attention and speech perception of children with CIs.

There is also previous evidence that in the participants of the present study, singing is connected to enhanced development of cortical processing of changes in a musical stimulus in a multifeature paradigm. In the paradigm, two changes which are, based on functioning of CIs (see above), easy to detect with CIs (duration changes and gap insertions), and three which are (also based on the functioning of the CIs) difficult to detect with CIs (changes in pitch, musical instrument timbre, and intensity) were presented alternating with a piano tone standard stimulus (Torppa et al., 2014b). The stimulus rate was fast, mimicking the tempo in real music. During the follow-up of 14–17 months, the ERP-responses of “CI singers,” who sang regularly and whose parents sang for them extensively, and whose speech perception in noise was good (Torppa et al., 2018), had enhanced or rapidly developing P3a or P3a-like responses over all change types. In contrast, in “CI non-singers,” the P3a to changes in timbre became smaller and later over time (Torppa et al., 2014b). At the second time point of measurements, the P3a to two difficult-to-detect change types (F0 and timbre) was larger and earlier in the “CI singers” than in the “CI non-singers” (Torppa et al., 2014b,^2018^). In summary, in the previous studies with these same children, the amount of singing was associated with enhanced speech-in-noise perception, verbal skills, as well as increases in the cortical indices of automatic attention shifts (reflected in P3a responses) to those changes in musical stimuli which are difficult to detect with CIs. Therefore, investigating the possible impact of singing at home to the development of cortical processing of different changes in speech is the logical next step in unraveling the importance of home activities in the development of CI children’s auditory skills.

The present study aims to investigate the cortical processing and its development of speech changes in Finnish-speaking children with CIs and NH (from here on: CI and NH groups) who have participated in the studies of Torppa et al., 2014a,b, 2018, 2020. More specifically, two changes that are easy-to detect (gap insertions and duration changes) and three that are difficult to detect with CIs (vowel identity, pitch = F0, intensity) will be presented in the middle syllable of the pseudoword /tatata/, using a fast-rate multi-feature MMN paradigm (Partanen et al., 2011, 2013b), in which the standard and the deviants alternate in a pseudorandom fashion. The paradigm mimics the challenges posed by everyday speech perception and is also comparable to the previous paradigm with musical stimuli (Torppa et al., 2014b,^2018^). To get a more precise picture of differences across age groups, the children are further divided into preschoolers (4–6 year-olds) and schoolchildren (7–13-year-olds), as children go to school and learn to read at the age of seven in Finland. Acquiring reading skills is expected to affect the processing of speech changes in the present participants, as reading instruction is mainly focused on letter-to-sound correspondence due to the consistent orthography in Finnish (see, e.g., Korkeamäki and Dreher, 1993), leading to rapid acquisition of basic reading skills (see, e.g., Aro and Wimmer, 2003) and to increasing phonemic awareness (see, e.g., Lerkkanen et al., 2004). Thus, learning to read and write provides the children an opportunity to visually map the sounds and semantically relevant sound lengths of Finnish, and practice them intensively both by listening and producing speech. The effect of letter-sound mapping on cortical responses has been shown in the study of Lovio et al. (2012) in which active practice of letter-sound mapping with GraphoGame resulted in enhanced MMN responses. Naturally, overall cognitive development is also another possible factor affecting response sizes and polarities in preschoolers and schoolchildren, as discussed earlier in reference to the different ERPs. The decision to divide the age groups from age 6 years 9 months is, however, based on the likelihood of reading instruction enhancing cortical change detection in the children. In the current study, a longitudinal design is used, repeating the measurements after a 14–17 months follow-up period (similarly to Torppa et al., 2014a,b, 2018), and allowing the investigation of the development of cortical speech processing over time. Finally, to study the connections of ERP responses and their development to singing, the present study also investigates the links of ERP responses to children’s own and their parent’s singing for their children in the CI group.

Cortical processing is assessed with pMMR, MMN, P3a and LDN responses. Based on earlier findings, we expect that

(1) Overall, the children with CIs will show less mature cortical responses than their NH peers, and this will be pronounced for the difficult-to-detect changes compared to the easy-to-detect changes. Therefore, we expect that

(1a) For all change types, there will be more significant pMMRs (at group level) elicited at T1 and in the preschoolers than in the schoolchildren, and in the CI than the NH group, as it has been observed to diminish with maturation (Morr et al., 2002) and because the development of auditory cortex of the CI group is assumed to be delayed in maturation compared to the NH children due to the period of deafness before implantation (Campbell and Sharma, 2016);

(1b) Significant MMNs at group level will be elicited for all change types in both CI and NH groups at both time points (Vavatzanidis et al., 2015, 2016). However, they may be smaller in the CI group than in the NH group due to difficulties in auditory discrimination, and particularly for the difficult-to detect changes. In addition, MMN amplitudes will be smaller in preschoolers than in schoolchildren, and at T1 than at T2, for those changes that are coded in the Finnish writing system (vowels, vowel duration, and gap insertion), and for which discrimination is actively practiced when learning to read and write (Korkeamäki and Dreher, 1993; Suomi et al., 2008; Hämäläinen et al., 2015);

(1c) Significant P3a responses at group level are expected to be elicited for the easy-to-detect changes in both CI and NH groups at both time points. P3a may be smaller in the CI group than in the NH group due to difficulties in the discrimination of changes and attention shifting (Kileny et al., 1997; Limb and Roy, 2014);

(1d) Significant LDNs at group level will be elicited in the NH group, and especially for those changes that change word meaning in Finnish (vowel identity and duration changes, and gap insertions; Bishop et al., 2011; Kuuluvainen et al., 2016a). The LDNs will not be elicited in the CI group, as observed by Hu et al. (2021);

(2) In the CI group, singing at home (by the children themselves and/or by their parents) will enhance the children’s speech perception and change detection abilities (Torppa et al., 2014a,b, 2018), and will thus be reflected in larger pMMR and/or MMN responses, and in improved attention shift reflected by the enhancement of P3a (Torppa et al., 2014b).

## Materials and methods

### Participants

The participants were 21 congenitally deaf, unilaterally implanted (CI; 9 boys) and 22 NH (11 boys) children, aged 4–13 years at the time of the first recording (Table 1). All children were native, monolingual Finnish-speakers and attended mainstream daycare or school. The children with CIs included in the study (the CI group) had their CI switch-on prior to age three years one month, had full insertion of the electrode, and more than six CI channels in use. Note that at the time the data was collected, it was assumed that the optimal time for cochlear implantation would be before the age of 3.5–4.0 years (see e.g., Kral and Sharma, 2012) and thus implantation after the age of 3 years 6 months was set as an exclusion criterion. The children with CIs had no diagnosed additional developmental or linguistic problems, and no re-implantation between the two measurements. Before the first measurement (T1), all children with CIs had been using their implants continuously for at least 30 months, and before the second (T2), at least for 46 months. Seventeen children with CIs used Cochlear, and four used Med-EL devices. Four CI children used an acoustic hearing aid (HA) in the non-implanted ear. They listened the stimulus without the HA. Their thresholds for hearing in the non-implanted ear exceeded 50 dB at 250 Hz, 60 dB at 500 Hz, and 70 dB at 1000 Hz, and evidently none of them heard the stimuli with the non-implanted ear during the experiment (see Torppa et al., 2012). Moreover, based on auditory brainstem responses (ABR), the other participants did not have usable residual hearing in the non-implanted ear.

**TABLE 1:** Participant information.

Group	*N*	Age at T1/T2^1^	Gender (M/F)	Handedness (R/L)	Digit span (RP) T1^1^	Digit span (RP) T2^1^	Block design (SP) T2^1^
CI presch.	11	5 years 2 months (11 months)/ 6 years 5 months (8 months)	5/6	10/1	16.2 (6.0)	17.4 (6.3)*	9.7 (3.0)
NH presch.	11	5 years 2 months (8 months)/ 6 years 5 months (11 months)	6/5	10/1	20.5 (5.0)	23.8 (6.0)*	11.3 (3.0)
CI schoolch.	10	8 years 5 months (18 months)/ 9 years 8 months (18 months)	2/8	8/2	18.9 (7.1)*	22.2 (9.4)*	8.3 (2.8)*
NH schoolch.	11	8 years 8 months (23 months)/ 9 years 11 months (24 months)	5/6	11/0	27.1 (7.7)*	30.5 (7.1)*	10.6 (2.8)*

**Group**	***N***	**Age at switch-on of CI (mo)^1^**	**CI use prior T1 (mo)^1^**	**Etiology^1,2^**	**CI processor type^3^**	**Pure tone thresholds using CI** **(dB HL)^1,4^**	

CI presch.	11	18.6 (3.4)**	43.9 (8.3)***	6/5	9/2/0/0	26(19)/25(9)/28(9)/40(9)	
CI schoolch.	10	25.2 (6.2)**	73.7 (19.6)***	3/7	4/2/1/3	29(5)/26(6)/27(9)/34(7)	

*p < 0.05, **p < 0.01, ***p < 0.001. CI, cochlear implant; NH, normal hearing; T1/T2, time point 1/2; M, male; F, female; presch., preschoolers; schoolch., schoolchildren; RP, raw points; SP, standard points. ^1^Mean (standard deviation in brackets). ^2^Connexin 26/Unknown. ^3^Nucleus Freedom, implant type CIC4/Nucleus ESPrit 3G, implant type CIC3/Med-EL Tempo+/Med-EL Opus; coding strategy for Nucleus devices was ACE and for Med-EL, CIS. ^4^For 4,000 Hz/for mean of 500, 1,000, and 2,000 Hz/for 250 Hz/for 125 Hz.

The 22 NH children (the NH group) were siblings of the participating children with CIs or were recruited from local musical play schools, other ongoing studies at the University of Helsinki, or from the neighborhood of one of the authors. The CI and NH groups were matched as accurately as possible by age, gender, handedness and social and musical background. None of the NH children had any diagnosed developmental or linguistic problems, and their hearing was normal, as assessed in regular check-ups at child welfare clinics. Parents of the participants gave a written informed consent and the children gave their consent verbally.

The study was carried out in accordance with the Declaration of Helsinki and all procedures were approved by the ethical committees of the participating hospitals. All 43 children participated also in the Torppa et al., 2012, 2014a,b,^2018^, ^2020^ studies.

The children were divided into separate groups according to age. The age groups consisted of 11 CI and 11 NH preschoolers (under the age of 6 years 9 months) and 10 CI and 11 NH schoolchildren (over the age of 6 years 9 months; see Table 1). The CI vs. NH age groups (CI vs. NH preschoolers and CI vs. NH schoolchildren) did not differ statistically for age at T1 [*t*(20/19) = 0.068/–0.38, *p* > 0.05] or T2 [*t*(20/19 = 0.43/0.078, *p* > 0.05], time between measurements [*t*(20/19) = 0.093/–0.33, *p* > 0.05], gender [χ^2^(1) = 0.18/1.53, *p* > 0.05], handedness [χ^2^(1) = 0.000/2.43, *p* > 0.05], or mother’s education [χ^2^(1) = 1.49/0.40, *p* > 0.05]. At T1 and T2, the forward digit span task (Illinois test of Psycholinguistic Abilities; Kirk et al., 1974) was administered to assess the children’s verbal short term memory, and at T2, their non-verbal visuoconstructive performance was assessed with the block design task (Wechsler, 2010). For the CI vs. NH preschoolers, no group differences were observed for the digit span task at T1 [*t*(20) = –1.87, *p* > 0.05] or for the block design [*t*(20) = –1.19, *p* > 0.05] task, but by T2, the CI preschoolers performed worse than NH preschoolers in the digit span task [*t*(20) = –2.46, *p* = 0.023; See Table 1]. The CI schoolchildren performed worse than the NH schoolchildren in all tasks: at the digit span task at both T1 [*t*(19) = –2.55, *p* = 0.020] and T2 [*t*(19) = –2.23, *p* = 0.033], as well as in the block design [*t*(19) = –2.25, *p* = –036] task administered at T2. These age group differences in non-verbal intelligence (PIQ) and verbal short-term memory have been observed in the earlier studies of children with CIs (PIQ: Cejas et al., 2018; verbal short-term memory: Pisoni et al., 2011; Talli et al., 2018). It is also noteworthy that despite the group difference in the scores for block design, they were within the age-typical window of 8–12 standard points in all studied groups (Table 1), as expected by the previous studies on non-verbal intelligence of children with CIs (Cejas et al., 2018).

Comparing the CI preschoolers vs. CI schoolchildren, the CI preschoolers had had their implants switched on earlier than the CI schoolchildren by an average of 6.6 months [*t*(19) = 3.10, *p* = 0.006]. This is unsurprising regarding the aim to decrease the age of implantation in the generation of the present participants. However, there were no statistically significant differences for etiology [χ^2^(1) = 1.29, *p* > 0.05], processor type [χ^2^(3) = 5.89, *p* > 0.05], or pure tone hearing thresholds at 4,000 [*t*(19 = –0.58, *p* > 0.05], mean of 500, 1,000, and 1,000 [*t*(19) = –0.077, *p* < 0.05], 250 [*t*(18) = –0.78, *p* < 0.05], or 125 [*t*(13) = 1.37, *p* < 0.05] Hz dB HL, the last two measurements missing from 1 and 6 children, respectively.

The effect of singing on the ERPs was tested for the CI group. Since it was expected that both the singing by the children themselves and parental singing could play a role for brain responses and speech processing (Torppa et al., 2014a,b, 2018, 2020), we used here the data for both aspects of singing at home. Children’s own and their parents’ singing was investigated with questionnaires addressed to parents at T1 and T2. Parents indicated how often they sang with their child, and how often the child sang themselves before T1 and between T1 and T2 using Likert scale 0–5 (see Torppa et al., 2020). The data for both time points was connected by calculating the mean of the responses. To ensure that the parents were able to identify singing of the children with CIs as different from speech, we recorded the children’s singing of “Twinkle twinkle little star” at T2, and the recordings were assessed blindly by a teacher of singing. In conclusion, the children’s singing was recognizable and different from general speech (see Torppa et al., 2014a for further information). Although singing was investigated in CI children only, we would like to note that the parental singing scores for the CI vs. NH children did not differ between the groups [*t*(38) = –0.82, *p* > 0.05] (note that there was no information on parental singing for three NH children). There was no information on the NH children’s own singing, and hence that could not be compared between the groups.

### Stimuli and procedure

The naturally spoken trisyllabic pseudoword/tatata/ and its variants served as stimuli (see Table 2 and Supplementary material 1). The duration of the standard stimulus was 480 ms and it included two silent gaps of 60 ms between the approximately 120 ms long syllables (for details, see Partanen et al., 2011). In the deviants, the middle syllable varied with either vowel duration, F0, gap insertion, intensity, or vowel identity. The F0, intensity, and gap deviants were made by manipulating the standard stimulus with Praat (Boersma and Weenink, 2001; see also Partanen et al., 2011), and appeared in the beginning of the second syllable (198 ms after the stimulus onset). For the vowel duration deviant, the difference from the standard became apparent approximately 280 ms from stimulus onset. The F0, intensity and gap insertion deviants were derived from the standard stimulus by manipulating it in Praat (Boersma and Weenink, 2001). However, the vowel identity and duration deviants were produced naturally during the recording of the stimuli, and thus their first syllable differed slightly from that of the standard (see Supplementary material 1 for waveforms and spectrograms of the stimuli). Using natural stimuli preserved full information available in natural speech and allowed for comparison of speech processing with CIs vs. in NH in an ecologically valid setting. The F0 deviants had two magnitudes (increases of 15 and 50% in F0), and the intensity deviants were either 6 dB increments or decrements. By dividing these deviant types to two different difficulty levels, we wished to see if processing of these would be different for participating child groups. The stimulus onset asynchrony (SOA) was 900 ms.

**TABLE 2:** Stimulus information.

Stimulus	Syllables	Total duration (ms)	Gap between 1st and 2nd syllable (ms)	Middle syllable duration (ms)	Middle syllable F0 (Hz)	Middle syllable F1/F2/F3 (Hz)	Middle syllable intensity (dB SPL)
Standard	/tatata/	480	60	120	169	730/1476/2700	60/70
Gap	/tattata/	580	160	*std*	*std*	*std*	*std*
Vowel duration	/tata:ta/	560	*std*	200	*std*	*std*	*std*
Vowel identity	/tatota/	*std*	*std*	*std*	*std*	560/1240/2750	*std*
F0 15%	*std*	*std*	*std*	*std*	194	*std*	*std*
F0 50%	*std*	*std*	*std*	*std*	254	*std*	*std*
Intensity –6 dB	*std*	*std*	*std*	*std*	*std*	*std*	54/64
Intensity + 6 dB	*std*	*std*	*std*	*std*	*std*	*std*	66/76

std, same as in the standard stimulus; F0, fundamental frequency.

The stimuli were presented through two high-quality loudspeakers situated in an acoustically and electronically shielded room, in approximately 45° angles and 1 m distance from the subject’s ears on both sides of the subject. Sounds were presented at most comfortable level of 60 dB SPL for the NH and 70 dB SPL (excluding intensity increment deviants) for the CI group as measured from the ear cantus (for one child with a CI the level had to be lowered to 65 dB SPL because 70 dB SPL was uncomfortable for her). The sound intensities were higher for the CI group to accommodate for their higher thresholds for hearing in free field using their CIs (see Table 1) compared to those of normally hearing children (Rahko and Karma, 1989; Haapaniemi, 1996). During the 30-min EEG experiment, the children watched a silent movie and listened passively to the stimuli. In the one-block stimulus sequence standard and deviant syllables alternated so that the deviants occurred pseudorandomly in the sequences: repetitions of the same deviant type in excess of two were swapped with a deviant in random location of the sequence, after which the sequence was checked again for repetitions until no more such repetitions were present. In total, 2,000 stimuli were presented, with 1000 standard and 5 × 200 equiprobable different deviant stimuli, the F0 and intensity deviants having 100 of each type of change magnitude. The stimuli were presented twice (at T1 and T2) for the participants. The time between T1 and T2 was 14–17 months.

### Electroencephalography recording and data analysis

Electroencephalography recordings were conducted using Biosemi ActiveTwo amplifier and 64 active electrodes embedded in a cap (Biosemi B.V., Netherlands), with a sampling rate of 512 Hz and a recording band-pass filtering of DC-102.4 Hz. The CMS/DRL electrodes were used as an online reference. Additional electrodes were placed at the left and right mastoid, the nose, and at the canthi to record eye movements and blinks.

The EEG data were first analyzed using EEGLAB 8 (Delorme and Makeig, 2004). The data were downsampled at 256 Hz, and highpass filtered above 0.5 Hz, and re-referenced to the nose. Because of the location of the CI device, some channels could not be used, and data from these electrodes were interpolated. To remove ocular and muscle artifacts in both CI and NH groups, an independent component analysis (ICA) with the Fastica algorithm was applied (Makeig et al., 2004). In addition, ICA was used in the CI group to reduce the CI related artifact (see Näätänen et al., 2017; and Torppa et al., 2012 for the details of the procedure). Before ICA, data dimensionality was narrowed down by the amount of interpolated channels, and automatic epoch rejection at a threshold between ±300 and ±400 μV was performed. The rejection thresholds were individually adjusted to preserve at least 85% of original epochs for effective statistical analysis.

After ICA, the epoch voltage rejection was done again with a threshold of ±150 μV, using an epoch of 800 ms, starting 100 ms before stimulus presentation. Further, the proportion of remaining epochs after voltage rejection was analyzed for each individual subject. The minimum was set at 73% (73) of remaining epochs for each deviant, in order to preserve as many participants as possible, as this was the number of remaining epochs of one NH child for the intensity increment deviant, with his/her data otherwise being of good quality (78–96% of epochs preserved). On average, 182 (91%) epochs were preserved in the CI group and 188 (94%) in the NH group per condition. To increase the signal to noise ratio, a region of interest (ROI) including F3, Fz, F4, C3, Cz, and C4 electrode locations were averaged for the final ERPs (see Näätänen et al., 2017 for further information). Difference waveforms were calculated by subtracting standard ERP waveform from that of each deviant. This procedure was used in all ERP analyses.

Based on previous findings using the same paradigm (Partanen et al., 2013b) and visual inspection of the ROI ERP difference (deviant-standard) waveforms, peak latencies were determined for each group and time point as follows: First, the MMN was identified as the largest negative deflection at 300–450 ms from stimulus onset. As the second syllable, in which the changes occurred, began at 198 ms from stimulus onset (see Table 2), the MMN was assessed to be elicited earliest by 300 ms from stimulus onset (or 102 ms from the start of the second syllable). Then, the pMMR was identified as a positivity preceding the MMN, at a time window starting from 250 ms and ending at the identified MMN peak (i.e., the pMMR, if present, had to precede the MMN). Two hundred and fifty milliseconds from stimulus onset was kept as the absolute lower bound for pMMR elicitation, since previous studies have shown that deviance detection in adults can occur at the earliest at 30–40 ms after stimulus onset, around the adult P1 response (for reviews, see Escera and Malmierca, 2014; Shtyrov and Lenzen, 2017). Children’s P1 is elicited typically around 80–100 ms (see, e.g., Sharma et al., 2015; Kuuluvainen et al., 2016b), so the pMMR lower bound was set to 52 ms from second syllable onset. The P3a was identified as a positive deflection after the MMN, at a time window starting from the MMN peak and ending at 525 ms, and finally, the LDN was identified from a time window starting from the P3a peak (or MMN peak if the P3a was not observable) and ending at 650 ms. Thus, for each group, timepoint and deviant, the four responses, if elicited, were in the temporal order of pMMR-MMN-P3a-LDN. Out of these 224 responses (four groups, measured twice for 7 deviants, four responses per deviant) 21 could not be identified reliably. They were instead quantified either based on their peak latency at an electrode where the response was clearly peaking, or in three cases of pMMR, the lower bound of the time window at 250 ms was chosen for significance testing, as there was no visible peak within the time window in any electrode (see Table 3). The LDN had to be elicited by 650 ms from stimulus onset (or 452 ms post-change) for it to be included in the analyses.

**TABLE 3:** Response latencies.

		From stimulus onset (ms)								From the beginning of the 2nd syllable* (ms)							
			
		CI group				NH group				CI group				NH group			
					
		T1		T2		T1		T2		T1		T2		T1		T2	
									
Response	Deviant	Pr	Sc	Pr	Sc	Pr	Sc	Pr	Sc	Pr	Sc	Pr	Sc	Pr	Sc	Pr	Sc
pMMR	Gap	266	254	266	250^a^	281^a^	250	254	262	68	56	68	52	83	52	56	64
	Dur	246	266	270	289	301	297	293	301	48	68	72	91	103	99	95	103
	Vow	277	254	262	254	277	270	273	258	79	56	64	56	79	72	75	60
	F0 15%	293^a^	262	262	297	285^d^	254^d^	266	231^a^	95	64	64	99	87	56	68	33
	F0 50%	254	250	270	281	250	262^d^	289^d^	231	56	52	72	83	52	64	91	33
	Int –6 dB	281	254	254^b^	250	301^f^	242^c^	258	262	83	56	56	52	103	44	60	64
	Int + 6 dB	301	270	234^a^	250	246^f^	250	242	246	103	72	36	52	48	52	44	48
MMN	Gap	371	356	371	340	324	316	324	324	173	158	173	142	126	118	126	126
	Dur	395	379	402	402	387	383	387	395	197	181	204	204	189	185	189	197
	Vow	383	352	383	383	410	422	418	426^a^	185	154	185	185	212	224	220	228
	F0 15%	328	305	340	352	336	344	340	336	130	107	142	154	138	146	142	138
	F0 50%	387	375	422^e^	371	332	301^d^	348	375	189	177	224	173	134	103	150	177
	Int –6 dB	406	309	387^b^	406	441^a^	301^a^	414	402	208	111	189	208	243	103	216	204
	Int + 6 dB	391	340^a^	313^a^	301	430	481	324	406	193	142	115	103	232	283	126	208
P3a	Gap	453	465	488	457	445	441	445	445	255	267	290	259	247	243	247	247
	Dur	453	445	453	453	445	445	445	453	255	247	255	255	247	247	247	255
	Vow	430	438	441	441	449	504^a^	449	473^a^	232	240	243	243	251	306	251	275
	F0 15%	461	473	438	441	391^d^	383^d^	386^d^	453	263	275	240	243	193	185	188	255
	F0 50%	461	473	520	453	363	391^d^	391	406	263	275	322	255	165	193	193	208
	Int –6 dB	453	430	438^b^	449	527^a^	461^c^	453	477	255	232	240	251	329	263	255	279
	Int + 6 dB	520	410^a^	375^a^	348	465	516	391	441	322	212	177	150	267	318	193	243
LDN	Gap	547	535	559	535	543	543	539	539	349	337	361	337	345	345	341	341
	Dur	543	492	555	555	477	469	477	481	345	294	357	357	279	271	279	283
	Vow	570	535	578	578	551	551	543	547^a^	372	337	380	380	353	353	345	349
	F0 15%	598	523	586	574	430^d^	477^d^	520	488	400	325	388	376	232	279	322	290
	F0 50%	539	598	578	586	493	547^d^	574	500	341	400	380	388	295	349	376	302
	Int –6 dB	496	586	598^d^	574	566^a^	582^c^	539	570	298	388	400	376	368	384	341	372
	Int + 6 dB	570	582	594	586	547	551^a^	566	527^a^	372	384	396	388	349	353	368	329

CI, children with cochlear implants; NH, normal hearing; T1, first measurement; T2, second measurement; Pr, preschoolers; Sc, schoolchildren; Gap, gap insertion; Dur, Vowel duration change; Vow, Vowel identity change; F0, fundamental frequency change; Int, intensity change; pMMR, positive mismatch response; MMN, mismatch negativity; LDN, late differentiating negativity. *The middle syllable started approximately 198 ms from stimulus onset. For the duration deviant, the elongation becomes clearly apparent at 280 ms (Partanen et al., 2011). Note that for vowel identity and vowel duration deviants there are also small acoustic differences in the first syllable, as these deviants were produced naturally. Peak latency not clearly visible at ROI ERP so quantified at *^a^*Cz, *^b^*Fz, *^c^*F3, *^d^*C3, *^e^*F4, *^f^*C4 where peak was clearest. *^f^*Peaked before 250 ms, lower bound of time window at 250 ms chosen.

The data were quantified using EEGLAB 13.6.5b (Delorme and Makeig, 2004) from the ROI ERPs with the baseline set to zero during the 50 ms window before stimulus presentation onset, with an offline low-pass filter of 20 Hz. We used the median method as in Torppa et al. (2014b) in order to avoid including extreme values from possible overlapping noise due to the implants. In this method, the trials of each individual are grouped by stimulus type, and the median value of the signal amplitude values of one sample point is taken as representative of that sample point. Thus, the resulting curve from an individual consists of the samples having the median amplitude over the accepted trials.

Event-related potential amplitudes were quantified from difference waveforms of each deviant and the standard, using a 50 ms time window centered at the peak latency of each response in each of the four age groups, and for CI and NH groups separately (see Table 3).

### Statistical analyses

The significances of all ERP response amplitudes (hypothesis 1a) were tested with one sample t-tests comparing to zero. Responses in the CI and NH preschoolers and CI and NH schoolchildren were included in group comparisons if the response was significant at least one of the time points T1/T2 for at least one CI and at least one NH age group, at a *p* < 0.05 significance level (see Table 4). For the singing analysis, significances of the responses were tested in the entire CI group, combining the preschoolers and schoolchildren in this analysis (see Supplementary material 2). However, the ERP values based on age group quantification were used for significance testing, as the response latencies observed in the age groups were more likely to catch the individual responses better than the response latencies in the combined group. The association of the response with singing was analyzed if the response was significant (*p* < 0.05) in at least one of the time points T1 or T2.

**TABLE 4:** Response amplitudes.

		Response amplitudes for each subgroup at each timepoint (sd in brackets)							
		
		CI group				NH group			
			
		T1		T2		T1		T2	
					
Response	Deviant	Presch.	Schoolch.	Presch.	Schoolch.	Presch.	Schoolch.	Presch.	Schoolch.
pMMR	Gap	0.89(1.88)	1.98(1.82)**	–0.95(2.30)	0.61(1.43)	–1.37(1.61)*	–0.90(1.71)	–1.01(2.82)	–0.28(1.09)
	Duration	1.24(1.37)*	1.37(2.51)	0.84(1.68)	1.23(1.75)	0.48(3.06)	–1.63(1.78)*	–0.16(2.67)	–0.31(2.02)
	Vowel	0.90(1.92)	2.55(1.91)**	2.46(1.82)**	1.89(1.81)**	0.15(3.08)	0.45(1.90)	2.35(2.30)**	0.42(1.17)
	F0 15%	–0.18(1.37)	0.83(2.79)	0.40(2.18)	–0.57(2.14)	–1.84(3.38)	–0.44(3.57)	–0.19(2.17)	0.67(2.64)
	F0 50%	1.50(2.29)	0.02(3.44)	0.60(1.90)	–0.12(2.96)	–1.05(2.69)	–0.46(3.22)	–1.20(3.06)	–0.75(1.90)
	Intensity –6 dB	1.23(1.55)*	1.04(2.55)	0.93(2.65)	1.09(3.46)	–0.87(3.20)	–0.88(1.86)	–0.10(2.77)	0.10(2.55)
	Intensity + 6 dB	0.40(3.10)	0.34(2.64)	0.75(2.92)	1.68(3.06)	–1.02(3.79)	–0.71(2.54)	–1.01(2.82)	–0.14(1.77)
MMN	Gap	–1.85(1.23)***	–2.12(2.05)*	–1.76(2.00)*	–1.86(2.17)*	–4.08(1.94)***	–2.91(2.01)**	–4.84(2.54)***	–3.24(1.62)***
	Duration	–0.89(2.35)	–0.57(2.67)	–2.53(2.83)*	–2.48(1.81)**	–3.84(3.15)**	–4.62(2.25)***	–4.90(3.02)***	–3.86(2.64)**
	Vowel	0.00(1.64)	0.82(2.13)	0.59(2.05)	–1.31(1.83)	–2.77(4.00)*	–2.25(1.65)**	–1.73(3.21)	–2.38(2.32)**
	F0 15%	–0.33(1.19)	–0.11(2.33)	–1.68(2.56)	–1.46(3.16)	–2.66(3.35)*	–1.87(3.96)	–1.83(2.73)	–1.71(3.11)
	F0 50%	0.71(1.75)	0.11(2.88)	–0.29(2.43)	–0.51(3.21)	–2.70(2.72)**	–0.77(3.23)	–1.85(3.69)	–1.81(2.72)
	Intensity –6 dB	1.21(1.07)**	0.05(2.01)	1.15(3.64)	–0.17(3.57)	–1.82(3.91)	–1.19(2.20)	–1.64(3.39)	–1.06(1.37)*
	Intensity + 6 dB	–0.13(2.59)	–0.43(2.33)	–0.37(2.49)	0.89(3.16)	–2.75(4.84)	–2.30(3.17)*	–2.96(2.71)**	–1.46(2.81)
P3a	Gap	1.25(2.17)	3.56(2.89)**	1.88(3.05)	3.69(3.06)**	0.64(2.95)	1.96(2.72)*	0.96(2.46)	1.49(1.51)**
	Duration	1.17(1.63)*	1.09(2.56)	0.09(1.64)	–0.54(2.34)	–2.29(2.76)*	–2.76(2.31)**	–3.56(2.76)**	–1.90(2.12)*
	Vowel	1.28(2.12)	2.43(1.99)**	1.20(3.45)	–0.37(2.65)	–2.30(3.81)	–2.70(1.29)***	–1.58(3.47)	–2.55(2.26)**
	F0 15%	2.36(2.44)*	1.86(1.97)*	–0.32(2.20)	–0.57(3.10)	–1.69(3.76)	–1.65(3.47)	–1.30(3.65)	–0.98(2.01)
	F0 50%	2.49(3.27)	2.24(2.71)*	1.66(2.65)	1.11(2.88)	–2.58(2.28)**	–0.54(2.72)	–1.27(4.0)	–1.66(3.06)
	Intensity –6 dB	1.80(2.46)*	1.51(2.04)*	1.56(3.51)	0.21(4.98)	–0.63(3.42)	–0.89(2.18)	–0.94(2.80)	–0.35(2.34)
	Intensity + 6 dB	1.42(2.49)	–0.85(2.79)	0.62(2.40)	1.27(3.51)	–2.53(4.87)	–2.06(2.76)*	–2.00(2.99)	–1.36(2.51)
LDN	Gap	–2.29(1.47)***	–1.28(1.53)*	–1.39(2.43)	–0.54(1.89)	–1.65(3.00)	–0.48(2.15)	–2.16(2.91)*	0.01(2.41)
	Duration	–0.34(1.80)	–0.75(2.61)	–0.90(2.22)	–1.11(3.23)	–3.02(3.29)*	–2.97(2.16)**	–4.11(3.11)**	–2.12(2.21)*
	Vowel	–0.42(2.18)	–0.03(2.14)	–0.22(3.47)	–2.10(2.76)*	–3.58(3.20)**	–2.68(1.84)**	–3.04(1.82)***	–2.47(1.78)***
F0 15%	1.20(2.37)	0.96(1.83)	–0.03(3.07)	–1.20(3.13)	–1.95(3.66)	–1.53(3.28)	–2.18(3.90)	–1.00(2.95)	
	F0 50%	1.76(2.24)*	0.74(3.28)	0.22(3.13)	–0.43(3.82)	–3.60(2.32)***	–2.38(2.95)*	–3.23(2.90)**	–3.20(3.68)*
	Intensity –6 dB	1.46(2.18)	0.13(2.90)	0.04(3.73)	–0.77(3.87)	–1.68(3.72)	–1.85(2.37)*	–1.60(3.40)	–1.38(2.05)*
	Intensity + 6 dB	0.56(3.03)	–0.79(2.23)	0.41(3.11)	–1.15(3.21)	–2.44(5.20)	–2.30(3.67)	–3.01(3.09)**	–2.12(2.74)*

Response significance: *p < 0.05; **p < 0.01; ***p < 0.001. CI, children with cochlear implants; NH, normally hearing children; T1/T2, time point 1/2; Presch, preschoolers; Schoolch, schoolchildren; pMMR, positive mismatch response; MMN, mismatch negativity; LDN, late differentiating response; Gap, gap insertion; vowel, vowel identity change; duration, vowel duration increment; F0 15% and 50%, percentual changes of pitch; intensity ±, intensity increments and decrements. Responses in LMM group comparisons are highlighted in gray. Statistically significant responses with opposite polarity expected are in italics.

Linear mixed modeling (Singer and Willett, 2003; West, 2009) was used in statistical testing of (i) group differences of CI vs. NH in the age groups of preschoolers and schoolchildren (hypotheses 1b-d), and (ii) for the analysis of the effect of singing in the combined CI group (hypothesis 2; see also Torppa et al., 2014b,^2018^). The method was selected because it allows one to combine measurements from the several groups and two time points in a single analysis, and since it allows for missing data (Singer and Willett, 2003; West, 2009). The LMM was first estimated for fixed main effects of clinical group (CI vs. NH), age group (preschoolers vs. schoolchildren) and development over time (the difference between responses at T1 and T2) and all two- and three-way interactions, with participant as the random effect. In the second phase, in all LMM analyses, statistically not significant three-way interactions, and in the third phase, statistically not significant two-way interactions were removed, yielding a final model including the significant fixed effects and interactions, with participant as the random effect. The models were estimated with the aforementioned procedure separately for each response fulfilling the response significance inclusion criteria (see Table 4). Statistically significant interactions were further investigated with *post hoc* tests, using the Bonferroni correction for multiple comparisons.

Before testing hypothesis 2, the correlations of CI group’s singing scores and child age at T1 were analyzed, in order to determine if age should be included as an additional covariate. This was done as it is feasible to assume that preschoolers might sing more than schoolchildren, and parents might also sing more to preschoolers than schoolchildren. Correlations between the two singing scores, and between each score and age at T1 were not significant. Therefore, to test hypothesis 2, LMMs were estimated separately for both singing scores (parental and by the children themselves), and only the main effects of singing scores and time were included in the model as fixed effects and participant as the random effect. Including the singing scores in both fixed main effects and as a covariate allowed us to investigate the overall impact of singing on response amplitudes, as well as the interaction of measurement time and the singing scores. As in the previous LMM (to test hypotheses 1b–d), statistically not significant two-way interactions were removed from final models. Only the significant (*p* < 0.05) results related to the hypotheses are reported. All statistical analyses were made with SPSS 25 software (IBM Corp., Armonk, NY, USA).

## Results

### pMMR, MMN, P3a and LDN to (with CIs) difficult-to-detect changes in vowel duration and gap insertions between syllables

#### pMMR

**FIGURE 1:**
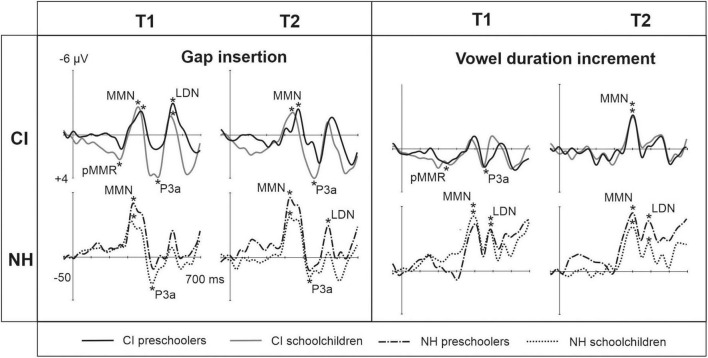
Difference waveforms (ERPs of deviant minus standard) from the six-electrode ROI (F3, Fz, F4, C3, Cz, C4) for the easy-to-detect change types of gap insertion and vowel duration at measurement time points T1 and T2 for the preschoolers (age below 6 years 9 months) and schoolchildren (age above 6 years and 9 months) with CI and NH. The data is referenced to the nose. Significant responses are marked with an asterisk. ERP, event related potentials; CI, children with cochlear implants; NH, normally hearing children; PR, preschoolers; SC, schoolchildren; pMMR, positive mismatch response; MMN, mismatch negativity; P3a, positive ERP following MMN; LDN, late differentiating negativity.

At T1, CI preschoolers had significant pMMRs to vowel duration changes, and CI schoolchildren to gap insertions. The responses were no longer significant at T2 (see Table 4 and Figure 1). No statistical group comparisons could be conducted, as the NH group did not have significant pMMRs to these deviants. Instead, the NH children had significant early negative responses at T1 in the pMMR time window: the preschoolers to gap insertions, and the schoolchildren to duration changes. By T2 these responses were no longer significant (see Table 4 and Figure 1).

**FIGURE 2:**
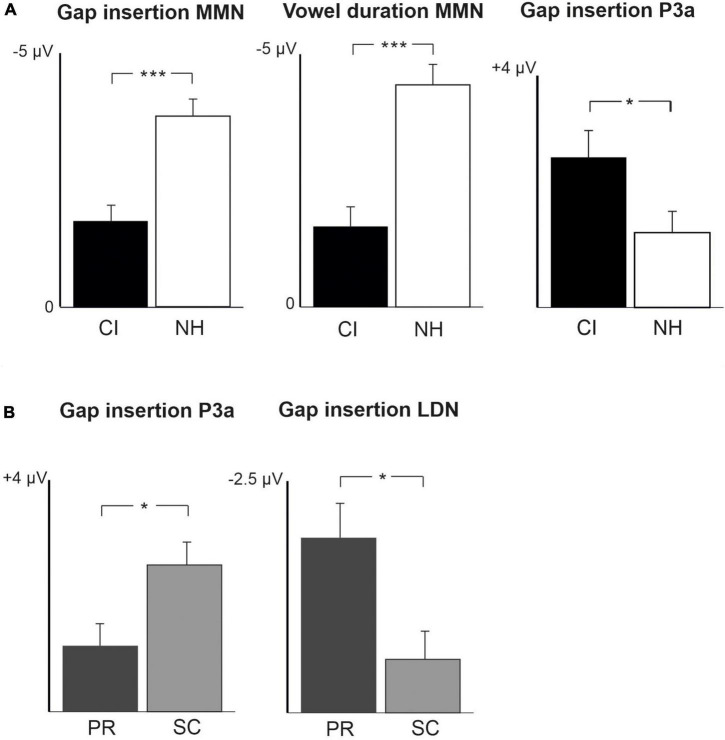
Barcharts of significant main effects and interactions in Linear Mixed Modeling for the easy-to-detect change types of gap insertion and vowel duration. **(A)** CI vs. NH group main effects; **(B)** age (preschoolers vs. schoolchildren) main effects; MMN, mismatch negativity; CI, cochlear implant; NH, normal hearing. *p < 0.05, **p < 0.01, ***p < 0.001. Note the different scales for different responses.

#### MMN

At the MMN time window the response polarities between groups were negative for all groups at both time points (Table 4 and Figure 1), and also statistically significant except for the MMN to vowel duration changes in both CI age groups at T1. Thus, the MMN responses for changes in vowel duration and gap insertion were both included in the statistical analyses of MMN (Table 4). Both MMNs were smaller for the CI group than for the NH group [gap: *F*(1,40) = 17.3, *p* < 0.001; duration: *F*(1,40) = 17.5, *p* < 0.001; Table 5 and Figure 2A].

**TABLE 5:** Estimates of fixed effects, standard errors, *t*-tests and confidence intervals in the LMM.

Response	Parameter	Estimate	*SE*	*df*	*t*	*P*-value	95% confidence interval	
							
							Lower bound	Upper bound
GAP MMN	CI vs. NH group	1.888	0.454	40	4.16	<0.001	0.971	2.806
DUR MMN	CI vs. NH group	2.689	0.643	40	4.18	<0.001	1.389	3.990
GAP P3a	CI vs. NH group	1.313	0.600	40	2.19	0.035	0.100	2.525
GAP P3a	Age group	–1.485	0.600	40	–2.48	0.018	–2.698	–0.273
GAP LDN	Age group	–1.308	0.495	40	–2.64	0.012	–2.308	–0.308
VOW pMMR	CI vs. NH group	1.092	0.498	40	2.19	0.034	0.085	2.098
VOW pMMR	Age group	1.260	0.628	75	2.01	0.048	0.010	2.511
VOW pMMR	Age group × time	–2.210	0.764	41	–2.89	0.006	–3.754	–0.667
**Singing (CI children only)**								
VOW pMMR	P-singing × time	–1.090	0.449	19	–2.42	0.025	–2.029	–0.150
VOW P3a	P-singing × time	–1.125	0.516	19	–2.18	0.042	–2.204	–0.046

LMM, linear mixed modeling; SE, standard error; df, degrees of freedom; GAP, gap insertion; DUR, duration increment; VOW, vowel identity change; MMN, mismatch negativity; LDN, late differentiating negativity; pMMR, positive mismatch response; CI, children with a cochlear implant; NH, normally hearing children; P-singing, parental singing.

#### P3a

For the gap insertion, significant P3a responses were found at both time points T1 and T2 for both CI and NH schoolchildren but not for CI nor NH preschoolers (Table 4 and Figure 1). For changes in vowel duration, only CI preschoolers had significant, positive P3a responses at T1 (Table 4). Notably, for the NH groups, responses were significant at the P3a time window, but negative in polarity for both time points T1 and T2. Thus, only the P3a to gap insertion was included in the group comparisons of P3a (Table 4). The P3a was larger for the CI group than for the NH group [*F*(1,40) = 4.79, *p* = 0.035; Table 5 and Figure 2A). There was also a main effect of age group [*F*(1,40) = 6.13, *p* = 0.018; Table 5 and Figure 2B]. Across CI and NH groups, the P3a was larger for schoolchildren than for preschoolers.

#### LDA

For gap insertions, LDNs were negative and significant for CI preschoolers and schoolchildren at T1, and for NH preschoolers at T2, but not significant for NH schoolchildren any time point. Instead, for changes in vowel duration, the LDNs were significant and negative for both NH age groups at both time points (Table 4 and Figure 1) while not significant for neither CI group at neither time point. Thus, only the LDN to gap insertions was included in group comparisons (Table 4). There was a main effect of age group [*F*(1,40) = 7.00, *p* = 0.012]. Across CI and NH groups, and as opposite to the results for gap P3a, the LDN was larger for preschoolers than for schoolchildren (Table 5 and Figure 2B).

### pMMR, MMN, P3a, and LDA to (with CIs) difficult-to-detect changes in vowel identity, pitch (F0) and intensity

#### pMMR

**FIGURE 3:**
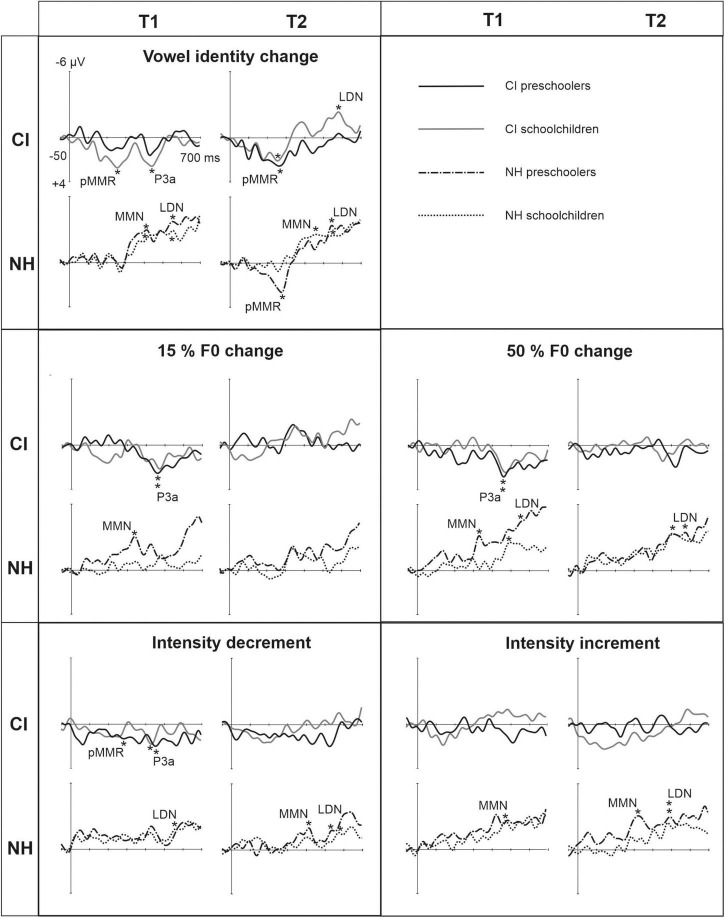
Difference waveforms (ERPs of deviant minus standard) from the six-electrode ROI (F3, Fz, F4, C3, Cz, C4) for the difficult-to-detect change types of vowel identity, F0 and intensity changes at measurement time points T1 and T2 for the preschoolers (age below 6 years 9 months) and schoolchildren (age above 6 years and 9 months) with CI and NH. The data is referenced to the nose. Significant responses are marked with an asterisk. ERP, event related potentials; CI, children with cochlear implants; NH, normally hearing children; pMMR, positive mismatch response; MMN, mismatch negativity; P3a, positive ERP following MMN; LDN, late differentiating negativity; F0, fundamental frequency.

**FIGURE 4:**
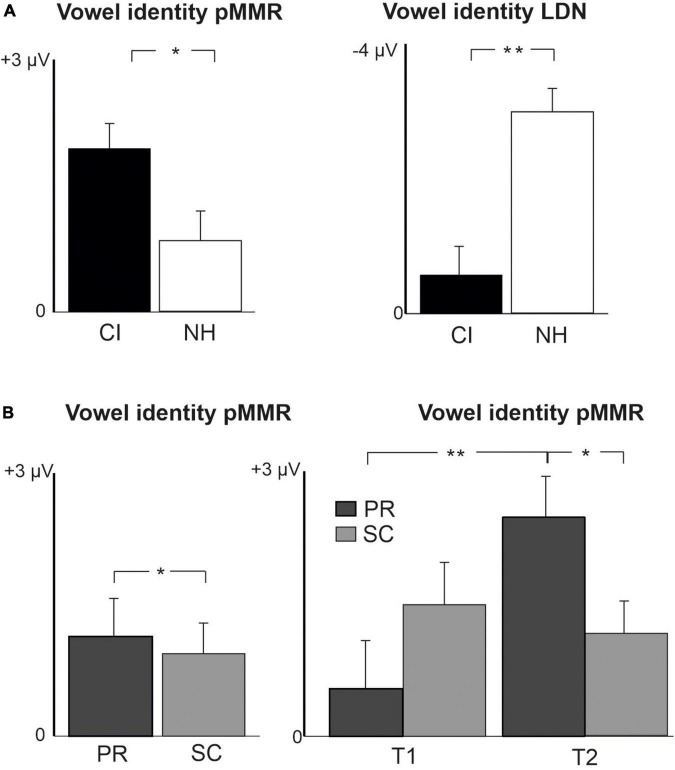
Barcharts of significant main effects and interactions in Linear Mixed Modeling for the difficult-to-detect change types of vowel identity, F0 and intensity changes. **(A)** CI vs. NH group main effects; **(B)** age (preschoolers vs. schoolchildren) main effects and age (preschoolers vs. schoolchildren) and time (measurement 1 and measurement 2) interactions; pMMR, positive mismatch response; LDN, late differentiating negativity; CI, children with cochlear implants; NH, normally hearing children; PR, preschoolers; SC, schoolchildren. *p < 0.05, **p < 0.01. Note the different scales for different responses.

For changes in vowel identity, significant pMMRs were elicited in CI preschoolers at T2, in CI schoolchildren at T1 and T2, and in NH preschoolers at T2 (Table 4). A significant pMMR to intensity decrements was elicited in the CI preschoolers at T1 (Table 4 and Figure 3). It was possible to include only the pMMR to changes in vowel identity in the statistical group comparisons (see Table 4). The pMMR of the CI group was larger than that of NH group [*F*(1,40) = 4.81, *p* = 0.034; Table 5 and Figure 4A]. In addition, across CI and NH groups, the preschoolers had larger pMMRs than schoolchildren at T2 [*F*(1,40 = 4.15, *p* = 0.048; Table 5 and Figure 4B]. A further interaction of age group and time [*F*(1,41) = 8.36, *p* = 0.006] resulted from (a) pMMRs in the preschoolers being smaller at T1 than at T2 (*p* = 0.001) whereas there was no statistically significant difference between T1 and T2 for the schoolchildren (*p* > 0.05) and (b) from preschoolers having larger pMMRs than schoolchildren at T2 (*p* = 0.048) but not at T1 (Table 5 and Figure 4B).

#### MMN

Statistical group comparisons could not be conducted for MMN responses to difficult-to-detect changes due to the complete lack of significant MMN responses in the CI group. Instead, a significant but positive response was elicited at the MMN time window in the CI preschoolers for the intensity decrement at T1. In contrast, at least one of the NH groups had a significant MMN to all deviant types at either T1 or T2 (Table 4 and Figure 3). More specifically, at T1, the MMNs were significant in the NH preschoolers for vowel identity changes and both F0 changes, and for the NH schoolchildren for vowel identity changes as well as intensity increments. At T2, MMNs to vowel identity changes were no longer significant in the NH preschoolers but were still significant in the NH schoolchildren. Furthermore, at T2, the NH preschoolers had significant MMNs to intensity increments and the NH schoolchildren for intensity decrements (Table 4 and Figure 3).

#### P3a

No significant P3a responses with correct polarity were elicited in the NH group to the difficult-to-detect changes (Table 4 and Figure 1). In contrast, one or both CI groups had at T1 significant P3a responses to all difficult-to-detect change types except intensity increments (Table 4). Significant P3as at T1 were elicited to the F0 15% and F0 50% changes as well as intensity decrements in the CI preschoolers, and to vowel identity, both F0 changes, and to intensity decrements in the schoolchildren. However, none of these responses were significant at T2 (Table 4 and Figure 3). In the NH children at T1, significant but negative responses were elicited in the P3a time window to F0 50% change in preschoolers, and to vowel identity and intensity increment changes in the schoolchildren. Statistical group comparisons could not be conducted due to lack of significant correct-polarity P3a responses in the NH group.

#### LDA

In the CI group, the only significant LDN response was elicited by the vowel identity changes in the CI schoolchildren at T2 (Table 4 and Figure 3). Notably, F0 50% change elicited only a significant positive response in the CI preschoolers at T1 in the time window for LDN. In the NH groups, significant LDN responses were elicited to all difficult-to-detect changes except F0 15% at some timepoint. Specifically, vowel identity and F0 50% changes elicited significant LDNs in both NH groups at both timepoints, the intensity decrement in NH schoolchildren at T1 and both NH age groups at T2, and the intensity increment in both NH age groups at T2 (Table 4 and Figure 3). Only the LDNs to vowel identity changes could be compared statistically between groups (Table 4). The LDN to vowel identity changes was smaller in the children with CIs than the NH children [*F*(1,40) = 13.1, *p* < 0.001; Figure 4A and Table 5].

### Children with cochlear implants and singing

Eighteen responses fulfilled the criteria of having the correct polarity and being significant (*p* < 0.05) in either T1 or T2 (or both) in the combined CI group (*N* = 21). These responses were the pMMRs to the gap insertion, duration increment, vowel identity change, and intensity decrement and increment, the MMNs to the gap insertion, duration increment, F0 15% change and intensity decrement, the P3a responses to the gap insertion, duration increment, vowel identity change, F0 15 and 50% changes, and intensity decrement, and LDNs to the gap insertion, and F0 15 and 50% changes (see Supplementary material 2 for amplitudes and *p*-values). Stable models could be estimated for both singing scores for pMMRs to duration increments, vowel identity changes, and the intensity decrement, for MMNs to vowel duration increment, and for P3as to the gap insertion, vowel identity changes, F0 15% changes, and intensity decrements. A stable model was also estimable for the LDN to F0 50% changes and the child’s singing score, but not the parental singing score. For the remaining responses the data was not sufficient for stable model estimation.

**FIGURE 5:**
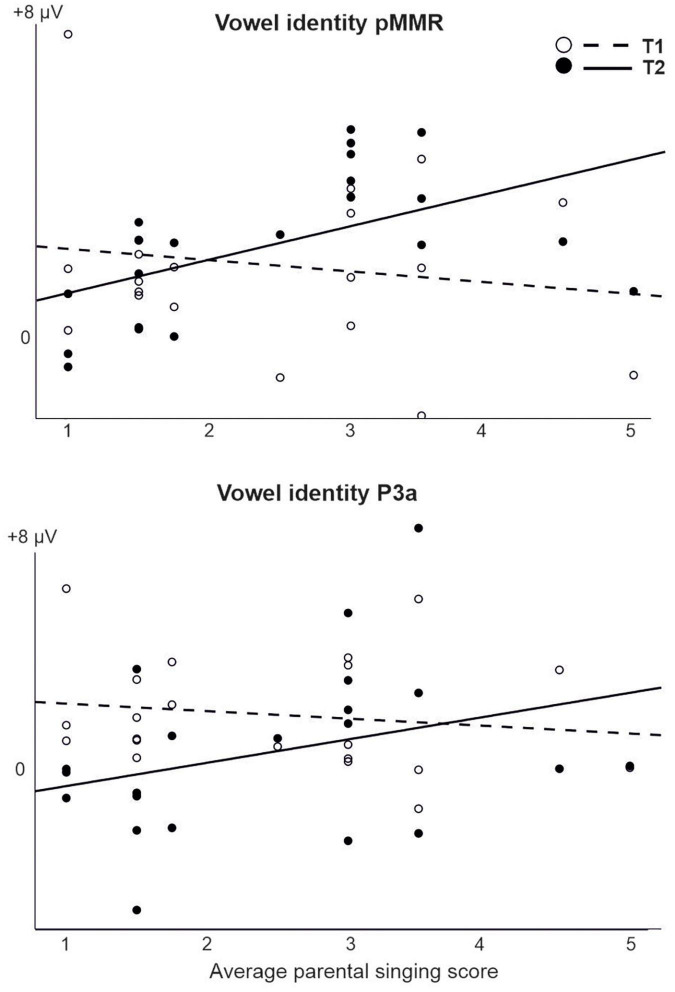
The interaction of parental singing and pMMR **(upper figure)** and P3a **(lower figure)** at time points of measurement T1 and T2 in the CI group as found in the LMM. Parental singing is a combination of the assessments by the parents themselves on a 1–5 Likert scale at T1 and T2. Note the intersection of the illustrative regression lines, which pinpoints the level of parental singing where the responses are approximately of equal size at T1 and T2. Thus, for the pMMR, singing increases the response size from T1 to T2 from an approximate singing level = 2, and for the P3a, singing sustains the size of P3a from T1 to T2 from an approximate singing level = 4.

For those models that were estimable, most yielded only significant main effects of time (repeating those observed in the CI vs. NH analysis, and thus not reported here) or no significant results at all. However, a significant interaction of time (T1 vs. T2) and parental singing was found for pMMR [*F*(1,19) = 5.89, *p* = 0.025] and P3a [*F*(1,19) = 4.76, *p* = 0.042] to vowel identity changes (Table 5 and Figure 5). Importantly, for the pMMR, the response sizes had clearly increased from T1 to T2 in those children whose parents sang for them average or above the mean of the amount of parental singing (see Figure 3A; regression lines meet at parental singing level ≈ 2). However, for the P3a, less parental singing was seen as a diminishing P3a from T1 to T2, whereas in those children whose parents sang more for them, the P3a amplitudes remained at about the same level between T1 and T2 (see Figure 5; regression lines meet at parental singing level ≈ 4).

## Discussion

The current study compared, to the best of our knowledge, for the first time the development of all four cognitive ERP responses pMMR, MMN, P3a and LDN to changes in pseudowords between children with CIs and their NH controls, aged 4–13 years. The ERP responses were recorded twice, 14–17 months apart (at T1 and T2). Children were divided into preschoolers (under the age of 6 years 9 months) and schoolchildren (over the age of 6 years 9 months). The differences in the development of responses between the children with CIs and NH were most evident for the change types which are, based on the previous findings and functioning of the CIs, difficult to perceive with CIs (F0, intensity and vowels; see Drennan and Rubinstein, 2008; Limb and Roy, 2014). Overall, the results were rather similar to those from the previous studies using musical stimuli (Torppa et al., 2012, 2018, 2014b). Importantly, the development of the pMMR and the P3a responses to changes in vowel identity were linked to parental singing in children with CIs, consistent with the findings from Torppa et al. (2018).

### Event-related potential responses and their development for the easy-to-detect changes

In line with the hypothesis 1a, the pMMR was not elicited to the easy-to-detect change types in the NH group. At T1 (the first time point of EEG recordings), the pMMR was found to changes in vowel duration in the CI preschoolers, and to gap insertions between syllables in the CI schoolchildren, both responses not being significant at T2. Thus, the results implicate that while the auditory system of children with CIs registered the changes in duration and gap insertions between syllables, the processing of these changes was immature at T1, but no longer at T2, as the pMMR is a response typically seen only in young children (Morr et al., 2002; Shafer et al., 2010). Moreover, the pMMR to gap insertions might be a sensitive marker the state of cortical maturation of speech processing in later-implanted children aged between 6 years 9 months and 13 years.

Hypothesis 1b of significant MMN responses for the easy-to-detect changes in both CI and NH groups was supported. For both CI and NH age groups, the MMN responses to gap insertions were elicited at both time points of measurements, and this was the case also for the MMN responses to changes in duration in the NH group. However, the MMN to vowel duration changes was significant for both CI age groups only at T2 (see Table 4). Thus, the appearance of MMN to duration changes at T2 in the children with CIs might be related to the maturation of cortical processing and related brain networks since simultaneously, pMMR to changes in duration disappeared at T2. It is possible that when cortical processing of children with CIs is unmature, discrimination duration changes is reflected in their pMMR responses. However, when cortical processing matures, the discrimination is reflected in their MMN responses. This is in line with the previous findings, showing development of the pMMR toward a MMN in young children with NH (see, for instance, Shafer et al., 2010).

Also supporting the hypothesis 1b, the MMNs to both easy-to-detect change types were smaller in the CI group than in the NH group, suggesting poorer sound discrimination for children with CIs (see Näätänen et al., 2017). Thus, there is poorer cortical discrimination of these changes despite the fact that CIs can deliver these gross temporal changes rather well. It is possible that the presence of pMMR responses in the CI participants decreased their MMN responses to the extent that the MMN was found to be smaller in statistical testing. Unfortunately, the children could not be tested behaviorally for their ability to discriminate the stimuli due to their young age (the youngest were 4 years old), and thus we do not know to what extent the differences in their cortical responses affect their discrimination performance. However, the present results are consistent with the findings in Torppa et al. (2012) with the same participants as in the present study. When the children were presented musical stimuli with a fast tempo mimicking real music, the MMN to gap insertions peaked later, and the MMN to changes in duration was smaller and later for children with CIs than for children with NH, suggesting poorer discrimination of these changes in the CI group. For the gap changes, this can be explained by simultaneous spectral and amplitude cues making gap detection difficult with CIs, as Sagi et al. (2009) proposed. They found poorer gap detection for postlingually deafened English-speaking adults with CIs than for NH controls when they identified gaps in synthesized speech stimuli. Furthermore, the fast tempo of the present multifeature paradigm could also make both duration change and gap insertion detection difficult for children with CIs.

Interestingly, and supporting hypothesis 1c, significant P3a responses to gap insertions were found at both time points for the schoolchildren (but not for the preschoolers) in both CI and NH groups, suggesting that these changes were distractive and captured the attention of schoolchildren only. Moreover, against hypothesis 1c, and despite the smaller MMNs to gap insertions, the gap P3a was larger in the CI than the NH group, and larger in schoolchildren (combining CI and NH groups) than in preschoolers (combining CI and NH groups). The larger P3a for gap insertions in the CI group may be related to the late CI activation of the CI schoolchildren participating the present study. It is known that later implantation leads to higher reliance in visual and tactile stimuli (for a review, see Glick and Sharma, 2017). This might further lead to higher reliance on reading and writing in the development of processing of sound changes affecting word meaning in school-aged, later-implanted children with CIs.

Overall, as assumed in our hypothesis 1b, schoolchildren in both groups have possibly learned to pay more attention to silent gaps between syllables since they have had to learn to distinguish gap insertions with reading and writing [in Finnish, semantically relevant gap insertions are spelled with double letters, distinguishing, e.g., between taka (hind) and takka (fireplace)]. Thus, learning to read and write could lead to stronger attention shifts toward gaps between consonants, especially as it is an audiovisual exercise and thus gives additional cues as to where to expect gaps in Finnish speech. The interpretation on the effect of reading instruction is supported also by the results of Engström et al. (2020). They found that in some children with CIs, multisensory learning with GraphoGame, in which children learn to map speech sounds to letters, led to a change from negative to positive ERP responses. Finally, singing, which is also a multisensory activity, was associated with enhanced P3a responses and their development in the study by Torppa et al. (2014b). Thus, there is a possibility that multisensory learning led to more efficient attention call toward gap insertions reflected in P3a.

In line with hypothesis 1d and previous findings from Uhlén et al. (2017) and Hu et al. (2021), the LDN to changes in vowel duration was elicited for the NH group (for both age groups and time points of measurements) while not for the CI group. Thus, as we expected, it seems that while for children with NH aged 4–13 years, the cortical maturation and accuracy of change detection allows further cognitive processing of duration changes, this is not the case for their peers hearing with CIs. However, again, the processing of changes in duration differed from that of gap insertions. Against our hypothesis 1d, the LDNs to gap insertions were elicited in both CI age groups at T1, and as we expected, in the NH preschoolers at T2, but not in NH schoolchildren at T1 or T2. Statistical group comparisons showed that across CI and NH groups, preschoolers had larger LDN responses than schoolchildren. These results suggest the possibility that while the LDN was still growing in preschool-aged children with NH, it was already disappearing in the school-aged children with NH. In line with the latter, previous findings on LDN have showed that LDN diminishes with age for the listeners who do not have problems with hearing or language processing, and is absent by adulthood (Bishop et al., 2011; Kuuluvainen et al., 2014, 2016a; Liu et al., 2014). The present results may indicate, similarly to the results for P3a for duration changes, that that the development is similar but later for children with CIs. Notably, the present findings are in accordance with the assumption that while the elicitation of the LDN is associated with immaturity of the cortex, possibly indexing a need for further processing, the immaturity disappears with age.

### Event-related potential responses and their development for the difficult-to-detect changes

We classified the changes in pitch (F0), intensity and vowel identity as difficult-to detect changes based on the previous findings and functioning of the CIs (Geers et al., 2003; Donaldson and Kreft, 2006; Drennan and Rubinstein, 2008; for a review, see Rødvik et al., 2018). Overall, for the CI group, all significant responses were positive except for the LDN response to changes in vowel identity at T2 in CI schoolchildren. Moreover, except for the pMMRs to vowel identity changes, for the NH group all significant responses to the difficult-to-detect changes were negative, emphasizing the crucial differences between these child groups.

In more detail, as we expected in our hypothesis 1a, and similarly to the easy-to-detect-changes, there were more significant pMMRs in the CI than the NH group. Surprisingly, for NH preschoolers, the pMMR to changes in vowel identity was significant only at T2. Surprising was also that, across CI and NH groups, for the preschoolers, the pMMR amplitude to vowel identity changes became larger by time. This contradicts with the findings that pMMRs changes to negative MMNs already at the age of 4–7 years (Morr et al., 2002; Shafer et al., 2010), and with the present findings indicating the disappearance of pMMR to changes in vowel duration and gap insertions between consonants in the children with CIs (see section “ERP responses and their development for the easy-to-detect changes”). Regarding the vowel identity changes, the previous findings are based on experiments where only one vowel change at a slow stimulus rate is presented (see, e.g., Shafer et al., 2010). In contrast, in the present study, vowel changes were presented with a fast stimulus rate, and they were embedded in the middle of a pseudoword, evidently making discrimination much more difficult than in the previous studies. All in all, it is thus possible that only for more difficult discrimination tasks such as the vowel identity discrimination in a sequence presented in a fast tempo, typical for speech in everyday life, the processing reflected by the pMMR matures in children with NH and CI only after the age of 13 years. However, further studies are needed to confirm our interpretations.

In line with our hypothesis 1b, MMN was elicited in children with NH to all difficult-to-detect change types by at least one age group at one time point. However, against our hypotheses, and contradicting with the present findings for the easy-to-detect changes, there was a complete lack of MMN to the difficult-to-detect changes in the children with CIs. Instead, in the time window for the MMN, most responses in the CI groups were not significant, the significant but *positive* response in the CI preschoolers to the intensity decrement at T1 being the only exception. The positivity of this response in the CI preschoolers in the time window of the MMN is consistent with the previous findings by Ortmann et al. (2013a,2017), Torppa et al. (2014a,2018), and Engström et al. (2020). One possible reason for the lack of significant responses to the intensity increments in the CI group is the activation of the automatic gain control (ACG) of the CI device (Stöbich et al., 1999). Based on the present and previous results on intensity increments, in future studies it might be better to use 60 dB–65 db SPL sound level for both child groups (children with CIs and NH) to avoid the effects of AGC.

Notably, when pMMR to changes in vowel identity appeared at T2 in the NH preschoolers, their MMN disappeared. This is in line with the findings that the polarity of mismatch responses to changes in vowels can vary in children for unknown reason (see Lee et al., 2012). Also the MMN to changes in F0 disappeared at T2 for NH preschoolers. Interestingly, this could be related to the relevance of F0 changes for younger children with NH, since F0 is the main auditory cue for sentence and word stress (see Torppa et al., 2014a), important especially for young children’s language learning (sentence stress: Männel and Friederici, 2013; word stress: Friedrich et al., 2009; Vavatzanidis et al., 2015) but not to the same extent any more for older children who already have good lexical skills (Mattys et al., 2005).

Moreover, against our hypothesis 1c, we found, at T1, P3a responses to difficult-to-detect changes only in children with CIs, more specifically to vowel identity changes, small and large changes in pitch (F0), and to intensity decrements – however, these responses were not significant any more at T2. In contrast, the NH children had significant but negative responses in the time window of the P3a, suggesting that their MMNs were prolonged to this time window, and possibly obscuring the P3as. We assume that the findings were related to interplay between increased listening effort in the CI groups, the consequences of that for activation of attention-related frontal areas of the brain, and the development of sound change discrimination and attention and related cortical networks.

It is known that the processing of degraded speech is highly dependent on auditory attention (e.g., Wild et al., 2012). Thus, when it is difficult to hear, listening becomes effortful and higher attention is needed for discrimination of speech. This has been shown for adults with hearing impairments, for whom increased listening effort leads to increased activation in cortical regions supporting executive function, attention, memory, and sensorimotor processing (Dimitrijevic et al., 2019). Moreover, for children with CIs, Ortmann et al. (2013a) found that when children with CIs showed positive responses in the MMN time window, their attention-related brain areas were activated very early. Thus, it is possible that the appearance of P3a responses at T1 and their disappearance at T2 only in children with CIs, reflects increase and decrease in listening effort. This interpretation is tentatively supported by the findings of Bertoli and Bodmer (2014), who found that in older adults with hearing loss, increases in the size of the P3a were associated with increased listening effort. The decrease in listening effort related to P3a would be consistent with the findings that the speech perception of children with CIs improves over time (Lee et al., 2002; Davidson et al., 2011; Dunn et al., 2014; Moein et al., 2017), evidently leading to less listening effort over time. Interestingly, listening effort could play a role also in the results for children with NH from Lee et al. (2012), showing that when speech stimulus contrasts were obviously harder to discriminate, positive mismatch responses were found especially in younger children. It should be noted that the responses in Lee et al. (2012) were elicited at around 300 ms, and were preceded by a small negative-going deflection at a similar latency to the MMN to the easier contrasts (see Figures 3–5 in Lee et al., 2012). This raises the question of whether the pMMRs in Lee et al. (2012) are more akin to the MMN or rather enhanced P3a responses. Disentangling the pMMRs and P3as in children with no MMNs is thus a question that warrants further research.

As we predicted (hypothesis 1d), for the NH groups, significant LDN responses were elicited to all difficult-to-detect change types and they were found at both time points of measurements for both age groups, although to the F0 deviant significant responses were elicited only to the 50% but not the 15% change. Thus, the finding was rather similar to the easy-to-detect changes, implying that for children with NH, cortical maturation, accuracy of change detection or relevance of the sound change allows further cognitive processing of changes in vowel identity, pitch (F0) and intensity. The LDN was elicited in the NH group also to several changes that did not elicit a significant MMN or P3a. However, the LDN did not show signs of disappearance in the school-aged children with NH, which is different from the present findings for gap insertions and previous findings for several other types of stimuli (Bishop et al., 2011; Kuuluvainen et al., 2014, 2016a; Liu et al., 2014). Thus, it is possible that the immaturity reflected in LDN disappears at a later age for changes in vowel identity, pitch (F0) and intensity than for the changes in duration and gap insertions. Moreover, it is possible that the auditory system of children with NH registers these changes as relevant and important until a later age than of gap insertions and duration changes, or behaves differently for syllables embedded in the middle of a word rather than for single vowels or syllables used in previous studies (Bishop et al., 2011; Kuuluvainen et al., 2014, 2016a; Liu et al., 2014). Interestingly, against our predictions based on findings from Hu et al. (2021), however in line with Singh (2005), at T2 LDN was elicited also in the CI schoolchildren to changes in vowel identity, even though the LDN was smaller for them than for children with NH. Thus, it seems that by age and time, the later cognitive processing of vowel identity changes develops in children with CIs – perhaps simultaneously with the developmental changes in discrimination of vowels or related cortical networks. Overall, further research is needed to understand the cognitive processes reflected by the LDN.

### Parental singing was linked to positive mismatch response and P3a responses in children with cochlear implants

For the pMMR to vowel identity changes, the response sizes clearly increased from T1 to T2 in those children whose parental singing was average or above. These results suggest that parental singing improved vowel discrimination. First, from the responses typically reflecting sound discrimination (pMMR and MMN), only pMMR to vowel changes was elicited in the children with CIs. Second, elicitation of early positive MMR has been found to be associated with good speech perception in children with Cis (Torppa et al., 2018; Engström et al., 2020). Furthermore, singing seemed to maintain the P3a response sizes between T1 and T2, while with less singing, the P3a diminished over time. The results were similar to the results from the earlier studies with the same children, which showed that only for the children with CIs who sang regularly (“CI singers”) and whose parents sang more for them, the P3a was maintained or increased over time (Torppa et al., 2014b,^2018^).

However, since in the present study, the direction of the connection of vowel identity pMMR and P3a to parental singing was similar, it is possible that here pMMR and P3 reflected similar processes for children with CIs. The responses of children with CIs were completely positive, and it is possible that the P3a responses were late mismatch responses or vice versa, the pMMR is related to both discrimination and attention. P3a has been interpreted to reflect evaluative discrimination related to the activation of an attentional switch mechanism (Friedman et al., 2001; Horváth et al., 2008), it is larger and earlier in CI children with better speech recognition (Kileny et al., 1997) and becomes larger with effective auditory training (Uther et al., 2006). Thus, it reflects both discrimination and attention shift toward sound changes. This assumption is consistent with those of Ortmann et al. (2013b), indicating based on an MMN source localization study that for children with CIs who perform well in speech discrimination, attention-related frontal areas are activated more than for poor performers. Importantly, even though in the comparisons of children with CIs and NH increased P3a responses are probably signs of increased listening effort, the increase in P3a across children with CIs does not mean less effective discrimination or attention functions. As opposite, since parental singing is related to better languages skills and speech perception (Torppa et al., 2018, 2020), those who have better attention functions as reflected in P3a responses (or pMMR responses) can discriminate better the degraded signal from CIs, inherently leading to reliance on attention in speech perception. This is particularly important for deaf-born children with CIs, for whom the spoken language should be brought directly to their attention, since due to difficulties in hearing, they cannot rely on passive listening or incidental language learning as efficiently as children with normal hearing (see e.g., Cole and Flexer, 2019).

We assume that the change in the strength of cortico-cortical connections as a result of parental singing could contribute to the present results. The neural network for P3a is distributed across frontal, parietal and temporal (auditory) cortical regions (Takahashi et al., 2013), suggesting functional connectivity between them. At an early age, the frontal, attention-related areas are developing in all children (Casey et al., 2000), and the increase in white-matter in association cortices, important for the maturation of auditory orienting, is already strong before the age of 8–12 months in normal-hearing children (Kushnerenko et al., 2013). It is evident that this development is delayed for deaf-born children with CIs since congenital deafness can also lead to degradation in white-matter volume in the auditory cortex and thus fewer afferent and efferent fibers (Emmorey et al., 2003). Parental singing, particularly at an early age, could improve the connections of auditory temporal areas to attention-related, frontal brain areas since singing of normal-hearing adults is related to enhanced connectivity between frontal and temporal cortical regions (Halwani et al., 2011; Wan et al., 2014), it is known that singing arises the attention of young children with CIs (Ronkainen, 2011), parental singing evokes and keeps the attention of young children more efficiently than speech (Politimou et al., 2019), and attention toward sounds modulates activation in auditory cortical areas (Fritz et al., 2007; Woods and Alain, 2009; Woods et al., 2009).

Overall, the results indicate, that singing face to face is good training of children’s vowel perception since it allows lipreading and a multisensory context for the perception of the formant changes associated with vowel changes (for a review, see Torppa et al., 2018). In accordance with this, Torppa et al. (2018) found that in children with CIs, more singing was associated with larger and earlier P3a responses for changes in timbre, for which spectral changes are important auditory cues similarly to changes in vowel identity (for a review, Torppa et al., 2018). Furthermore, Lo et al. (2020) found that musical training including singing improves perception of spectral resolution and speech in noise of children with hearing loss. Moreover, as discussed earlier, parental singing was in the present participants related to better speech perception and language skills (Torppa et al., 2018, 2020). As singing has no foreseeable negative consequences, and it carries benefits shown by previous studies, musical activities, including singing to and with the child, has been recommended as rehabilitation for children with CIs, and hearing impairments in general (Torppa and Huotilainen, 2019). Current results endorse this recommendation. However, more studies in other languages than Finnish are needed to pinpoint the possible benefits to speech perception across languages.

### Caveats and future directions

The present, mainly novel, results evoke many questions that call for answers in future studies. The sample size was small, and this was reflected as lack of statistical power in analysis for three-way interactions of clinical status, age group and time, and also regarding children’s own and parental singing, which was studied only in the children with CIs. However, it is hard to collect a large sample, not only in Finland but also in other countries, as mentioned in the review on studies on the relations of musical activities to children with CIs’s speech perception and language skills (Torppa and Huotilainen, 2019). It is clear that one should aim at larger sample sizes in the future studies related to cortical processing of speech, especially in children with CIs, possibly through joint efforts of different laboratories in different countries, and allowing also the disentangling of language-specific effects on cortical development in CI users.

The present participants were implanted with unilateral, old-generation CIs. Many of them were also implanted fairly late, some as late as at the age of three. It is known that bilateral and early implantation as well as new generation CI devices with new sound-processing technology lead to better speech perception performance (van Wieringen and Wouters, 2015; Hey et al., 2019; Warren et al., 2019). For instance, bilateral CIs allow listening with better ear, binaural summation, improved perception of speech in noise (if speech and noise sources are spatially separated), and localization of sounds, especially if the CIs are paired successfully (van Hoesel and Tyler, 2003; Litovsky et al., 2004; Hu and Dietz, 2015; van Wieringen and Wouters, 2015). These benefits are evidently important for good development of speech processing, and lack of these benefits can lead to poorer or different processing compared to bilaterally implanted children. Therefore, new research using similar paradigms are needed to assess the brain processing in today’s children with CIs, who are usually implanted at age of 1 or even before, are hearing with two CIs, and have new-generation devices. It would be especially valuable to study this group with methods better suited for source analysis, for example high-density EEG or optical imaging, which would allow pinpointing the neural processes underlying the different ERPs.

One possible issue is also the removal of the electric artifacts caused by the CI devices from the EEG signal. Even though ICA is a good method to eliminate the CI artifact from the auditory ERP responses when multichannel recordings are used as in the present study (Gilley et al., 2006; for a review, see Näätänen et al., 2017), ICA or residual artifact could affect the results between children with CIs and NH. We assume that this is not the case in the present study since the results between groups were clearly different for pMMR, MMN, P3a, and LDN, and between difficult- and easy-to-detect changes. Moreover, subtraction of responses to standards from responses to deviants was conducted which should eliminate the residual electric artifact from difference waveforms from which the responses were detected (Vavatzanidis et al., 2015). However, future studies are needed to compare ICA to other artifact elimination methods and to assess the role of other possible confounding aspects such as sampling rate or filtering in the evaluation of brain responses (see, for instance, Hu et al., 2015; Hu and Ewert, 2021; for other CI artifact elimination methods, see Wong and Gordon, 2009; Näätänen et al., 2017).

The present results also suggest that, over time, and with multisensory learning such as singing or learning to read and write, the sound change processing changes. However, in the current study, the impact of reading and writing instruction was only indirectly measured via the division of the children to preschoolers and schoolchildren. It is possible that the impact of overall cognitive development was more relevant to the observed changes in responses over time, as we know that the verbal short term memory as assessed with the digit span task increased between T1 and T2 (the increase is expected, as the score is available only in raw points and not standardized to age expectations due to the lack of up-to-date normative data for the test). However, the hypothesized impact of reading instruction is supported by the notion that the differences between preschoolers and schoolchildren emerged only for those changes which are relevant for accurate reading and writing in Finnish (gap insertions, vowel identity changes) and not to the changes in F0 or intensity. The interpretation is, in addition, supported by the results of Lovio et al. (2012) and Engström et al. (2020) using the GraphoGame intervention, which propose that reading instruction is indeed associated with changes in cortical sound processing in some children with CIs and NH.

Above all, future research is needed to confirm the interpretation that positive responses at the time window of MMN or preceding it in young children with CIs reflect early attention call or listening effort. More studies are also needed on how singing and visual or multisensory learning contribute to the development of ERP responses and underlying brain activity especially for children with CIs. It would also be important to study the children’s ability to discriminate the stimulus changes they hear in the ERP paradigm behaviorally, and thus gather data on how easy or hard the stimulus changes are to discriminate for both children with NH and CIs, taking into account the different challenges represented by different languages. Measuring the exact discrimination ability can be difficult if the participants are very young, but the present results reveal that the developmental changes in the brain processing of speech stimuli are evident starting from the age of four, and child-friendly behavioral discrimination paradigms could thus be very valuable. Finally, studies of whether the elicitation of the pMMR, MMN, P3a, and LDN at individual level is associated with the development of speech perception and discrimination, is another important area of study.

## Conclusion

The present results show that the development of ERP responses to natural, fast changes in pseudowords differs between children with CIs and NH especially for the difficult-to-detect changes with CIs (F0, vowel identity and intensity), while less difference between these child groups in the development was found for the easy-to-detect changes (vowel duration and gap insertion). The present findings on parental singing and developmental changes propose that cortical processing for the difficult-to-detect changes in children with CIs is associated with attentional processes. As listening effort has been previously shown to increase in adult CI users compared to NH (Dimitrijevic et al., 2019), and it has been associated with P3a size in a context different from the current study (Bertoli and Bodmer, 2014), the importance of listening effort in children with CIs and its relationship with cortical speech processing should be investigated further.

Our results, along those in Torppa et al. (2012, 2014b,^2018^, 2020), show that the multifeature paradigm is a useful tool to assess cortical processing of speech and music in children with CIs, and provides a wide range of information on different processes, when all responses (pMMR, MMN, P3a, and LDN) are analyzed, as in the current study. In the future, alongside with ERP studies, brain development in children with CIs should be studied using methodologies suited for localization of cortical and subcortical activity. Understanding the developmental trajectories and their associations with speech perception, attention, and listening effort, is highly relevant for the rehabilitation of children with CIs. It is also necessary to further investigate the impact of different types of multisensory training, such as singing or reading instruction, for the perceptual skills and brain processes in these children. Finally, it is important to encourage parents of children with CIs to sing, since singing can improve their children’s perception of speech.

## Data availability statement

The datasets presented in this article are not readily available because the data is sensitive. Requests to access the datasets should be directed to ritva.torppa@helsinki.fi.

## Ethics statement

The studies involving human participants were reviewed and approved by the Ethical Committee of HUCH and other participating hospitals (University Hospitals of Helsinki, Kuopio, Tampere, and Turku). Written informed consent to participate in this study was provided by the participants’ legal guardian/next of kin. The study was carried out in accordance with the Declaration of Helsinki.

## Author contributions

JL was responsible for statistical analyses. All authors listed have approved the work for publication.

## Abbreviations

CI: cochlear implant
EEG: electroencephalography
ERP: event-related potential
ICA: independent component analysis
LDN: late differentiating negativity
LMM: Linear Mixed Model
MMN: mismatch negativity
NH: normal hearing
pMMR: positive mismatch response
P3a: attention-related brain response peaking after MMN
T1 and T2: first and second time points of measurements.
