# Supplementary material for: The development of cortical processing of speech differs between children with cochlear implants and normal hearing and changes with parental singing
Source: Front Neurosci. 2022 Nov 18;16:976767. doi: 10.3389/fnins.2022.976767 (PMC9731313; doi:10.3389/fnins.2022.976767)
Supplement: Supplementary file 1 [file Data_Sheet_1.PDF]

Supplement 1

Standard sound

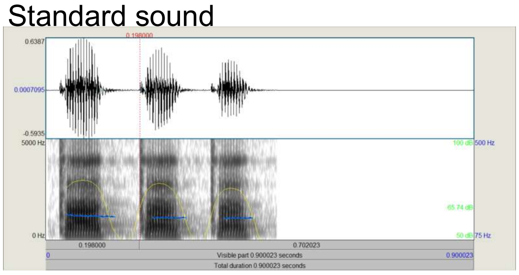

Standard sound

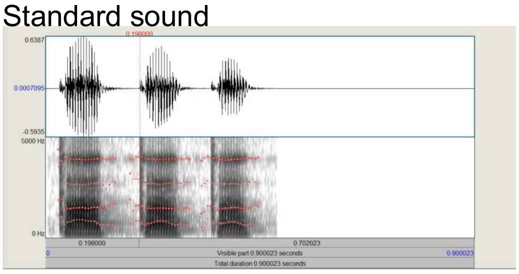

F0 15 % increment

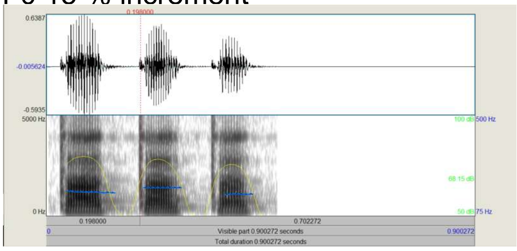

Vowel identity deviant

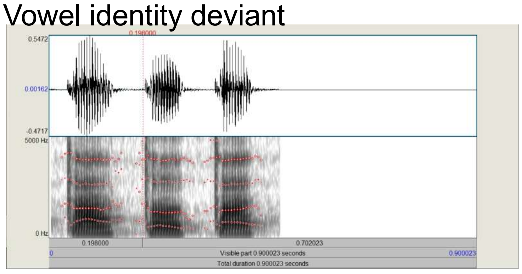

F0 50 % increment

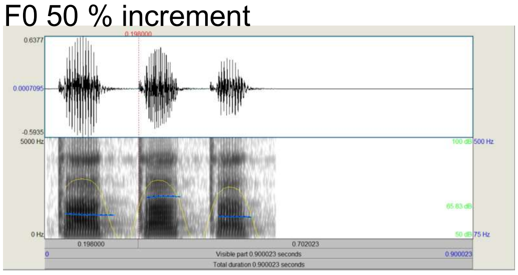

Vowel duration deviant

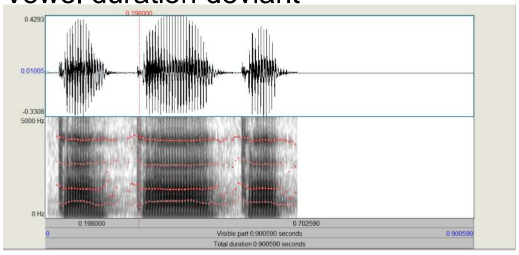

Intensity decrement

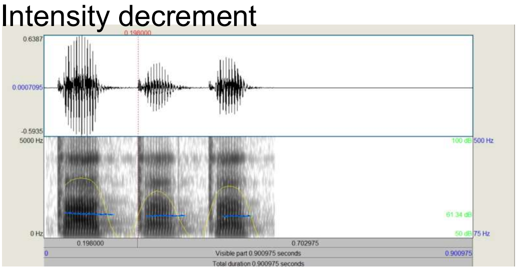

Gap insertion deviant

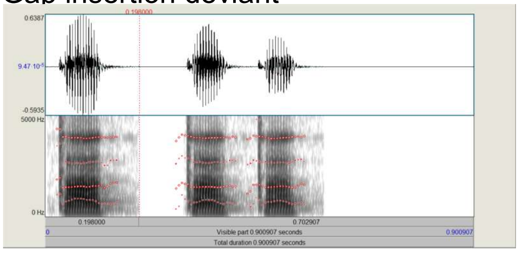

Intensity increment

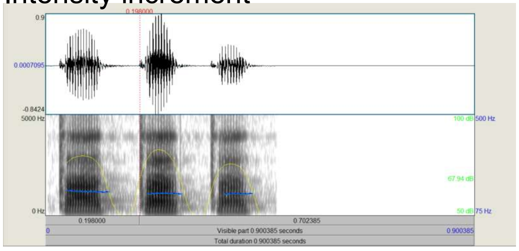

Supplementary Figure. Waveforms and spectrograms of the stimuli. The standard sound, vowel identity and vowel duration deviants were produced in the recording studio (note the small differences in the waveform of the first syllable). The F0 15 % and 50 % increment, intensity increment and decrement, and gap deviants were manipulated from the standard sound with Praat (see Partanen et al., 2011). The standard sound is shown on top row with pitch and intensity curves (left) and formant curves (right column). All sounds show a reference line at 198 ms, the approximate start of the second syllable.
