# Supplementary material for: The development of cortical processing of speech differs between children with cochlear implants and normal hearing and changes with parental singing
Source: Front Neurosci. 2022 Nov 18;16:976767. doi: 10.3389/fnins.2022.976767 (PMC9731313; doi:10.3389/fnins.2022.976767)
Supplement: Supplementary file 2 [file Data_Sheet_2.PDF]

Supplement 2: Response significances for the combined CI group

		Response amplitude (sd in brackets)	
		CI group	
**Response**	**Deviant**	**T1**	**T2**
**pMMR**	Gap	**1.41(1.89)****	-.21(2.05)
	Vowel duration	**1.30(1.94)****	**1.02(1.68)***
	Vowel identity	**1.68(2.05)*****	**2.19(1.79)*****
	F0 15 %	.49(2.13)	-.060(2.16)
	F0 50 %	.79(2.92)	.26(2.42)
	Intensity -6 dB	**1.14(2.03)****	1.01(2.99)
	Intensity +6 dB	.37(2.82)	**1.20(2.95)***
**MMN**	Gap	**-1.98(1.63)*****	**-1.81(2.03)*****
	Vowel duration	-.74(2.45)	**-2.50(2.14)*****
	Vowel identity	.39(1.88)	-.31(2.14)
	F0 15 %	.12(1.79)	**-1.58(2.76)****
	F0 50 %	.43(2.31)	-.40(2.79)
	Intensity -6 dB	**.66(1.66)***	.52(3.58)
	Intensity +6 dB	-.27(2.41)	.22(2.83)
**P3a**	Gap	**2.35(2.74)*****	**2.74(3.2)*****
	Vowel duration	**1.13(2.07)***	-.21(1.98)
	Vowel identity	**1.83(2.09)****	.45(3.98)
	F0 15 %	**2.13(2.19)*****	-.44(2.60)
	F0 50 %	**2.37(2.94)*****	**1.40(2.70)***
	Intensity -6 dB	**1.66(2.22)****	.92(4.22)
	Intensity +6 dB	-.34(2.82)	.93(2.92)
**LDN**	Gap	**-1.81(1.55)*****	**-.98(2.18)***
	Vowel duration	-.54(2.18)	-1.00(2.68)
	Vowel identity	-.23(2.11)	-1.12(3.22)
	F0 15 %	**1.09(2.08)****	-.56(3.08)
	F0 50 %	**1.27(2.76)***	-.082(3.40)
	Intensity -6 dB	.82(2.57)	.37(3.72)
	Intensity +6 dB	-.083(2.71)	-.34(3.19)

Response significance: *p<.05; **p<.01; ***p<.001; T1/T2 = time point 1/2

Responses taken into the singing rANCOVA analysis are highlighted in grey.
